# Remodeling of immune system functions by extracellular vesicles

**DOI:** 10.3389/fimmu.2025.1549107

**Published:** 2025-03-13

**Authors:** Deborah Neyrinck-Leglantier, Marie Tamagne, Raida Ben Rayana, Souganya Many, Marion Klea Pinheiro, Adèle Silane Delorme, Muriel Andrieu, Eric Boilard, Fabrice Cognasse, Hind Hamzeh-Cognasse, Santiago Perez-Patrigeon, Jean-Daniel Lelievre, France Pirenne, Sébastien Gallien, Benoît Vingert

**Affiliations:** ^1^ Univ Paris Est-Creteil (UPEC), Institut National de la Santé et de la Recherche Médicale (INSERM), Institut Mondor de la Recherche Biomédicale (IMRB), Creteil, France; ^2^ Etablissement Français du Sang (EFS), Ivry-sur-Seine, France; ^3^ Laboratory of Excellence, Biogénèse et Pathologies du Globule Rouge (GR-Ex), Paris, France; ^4^ Service de Maladies Infectieuses et Immunologie Clinique, Centre Hospitalier Universitaire Henri-Mondor, Assistance Publique-Hôpitaux de Paris (AP-HP), Université Paris-Est Créteil (UPEC), Créteil, France; ^5^ Institut Cochin, Inserm U1016, Centre National de la Recherche Scientifique (CNRS) UMR8104, Université Paris-Cité, Paris, France; ^6^ Faculté de Médecine and Centre de Recherche ARThrite, Université Laval, Québec, QC, Canada; ^7^ Centre de Recherche du Centre Hospitalier Universitaire de Québec-Université Laval, Québec, QC, Canada; ^8^ Etablissement Français du Sang Auvergne-Rhône-Alpes, Saint-Etienne, France; ^9^ Univ Jean Monnet, Mines Saint-Étienne, INSERM, U1059 Sainbiose, Saint-Étienne, France; ^10^ Division of Infectious Diseases, Queen’s University, Kingston, ON, Canada

**Keywords:** extracellular vesicles (EV), cellular remodeling, PLWH, cytokine secretion, immunomodulation

## Abstract

**Introduction:**

The treatment of chronic viral infections can often bring viral replication under control. However, chronic immune activation persists and can lead to the development of comorbid conditions, such as cardiovascular disease and cancer. This is particularly true for people living with HIV (PLWH), who have significantly more extracellular vesicles from membrane budding, also called plasma microparticles (MPs), than healthy individuals (HDs), and a much more immunomodulatory phenotype. We hypothesized that the number and phenotypic heterogeneity of MPs can trigger a functional remodeling of immune responses in PLWH, preventing full immune restoration.

**Methods:**

We investigated the rapid impact of three types of MPs — derived from membrane budding in platelets (CD41a^+^ PMPs), monocytes (CD14^+^ MMPs) and lymphocytes (CD3^+^ LMPs) in the plasma of PLWH or HDs—on four cell types (CD4^+^ and CD8^+^T lymphocytes, monocytes and DCs).

**Results:**

These investigations of the short multiple interactions and functions of MPs with these cells revealed an increase in the secretion of cytokines such as IFNg, IL2, IL6, IL12, IL17 and TNFa by the immune cells studied following interactions with MPs. We show that this functional remodeling of immune cells depends not only on the number, but also on the phenotype of MPs.

**Conclusion:**

These data suggest that the large numbers of MPs and their impact on functional remodeling in PLWH may be incompatible with the effective control of chronic infections, potentially leading to chronic immune activation and the onset of comorbid diseases.

## Introduction

Uncontrolled viral infections requiring treatment may lead to incomplete immune responses resulting in a functional defect of immune cells (1). HIV infection is the best known chronic viral infection. Some people living with HIV (PLWH), known as elite controllers, achieve natural control over the infection by generating an efficient polyfunctional and high-affinity response (2, 3). By contrast, the majority of PLWH are unable to achieve such control and require treatment with antiretroviral therapy (ART). ART controls viral replication, but cannot correct the excessive activation of the immune system in PLWH (4). This failure to regulate chronic immune system activation is associated with incomplete functional restoration of the immune system (4). Indeed, PLWH may have an immune system characterized by immunosenescence and premature aging (5–8), which can lead to an increase in the occurrence of often inflammatory comorbid conditions in PLWH (9–14).

The presence of extracellular vesicles (EVs) in the blood is thought to be one of the major elements underlying chronic immune activation (15–20). Various types of EVs exist, differing in size and cellular origin. Exosomes are small EVs (mean diameter between 40 and 200 nm) originating from intracellular compartments. Microparticles (MP), also known as ectosomes, are larger (mean diameter between 200 and 900 nm) and originate from plasma membrane budding. In this study, we focused exclusively on larger EVs, most likely MPs, as defined by the International Society for Extracellular Vesicles (21–23).

MPs are particularly interesting as they express many membrane and cytoplasmic proteins from their cell of origin — receptors, cell activation markers, immunomodulatory markers, cytokines, functional organelles and genetic material, for example — on their surface (17, 24–26). Most studies to date have not distinguished between subtypes of MPs according to the markers expressed on their surface, but these markers may provide important information about the modulatory role of EVs in the immune system.

MPs play a primordial role in intercellular communication and are able to modulate the immune system by interacting with diverse immune cells (conventional CD4^+^ T lymphocytes (TLs), Tfh, Th17, Treg, monocytes, B lymphocytes, dendritic cells) (20, 26–32).

EVs from red blood cell units can induce a pro-inflammatory response by promoting the secretion of cytokines such as IFNγ, IL2 and IL17 (28). The EVs present in platelet concentrates can alter macrophage differentiation and modulate the secretion of IFNγ, TNFα, IL6 and TGFβ by CD4 cells (27, 33). Platelet microparticles (PMPs) have also been shown to inhibit IL17 secretion by Tregs (29). In addition, we recently demonstrated the importance of CD41a^+^ PMPs for immune cell interaction and activation (34). The mechanisms by which MPs exert their effects remain incompletely understood, but the interactions between MPs and immune cells may involve immune ligands/receptors present on the surface of MPs. Indeed, we have recently shown that CD27^+^ and CD70^+^ MPs can increase the activation and lymphoproliferation of CD4^+^ TLs via receptor transfer (32). Furthermore, our most recent study showed that PMPs are responsible for major phenotypic and protein changes, leading to monocyte activation (34).

These immunomodulatory properties of MPs may shed light on the immune responses observed during the follow-up of PLWH. The role of MPs in HIV infection remains unknown, particularly in terms of their effects on immune activation, but there is a long-established link between the virus and MPs. Early studies of chemokine receptor transfer in HIV infection also highlighted the importance of studying EV interactions with immune system cells (35, 36).

We have shown that PLWH have particularly high levels of circulating MPs of all origins other than PMPs and monocyte microparticles (MMPs) (37). This finding does not necessarily imply the absence of a major role for PMPs and MMPs in immunomodulation.

We also observed a modification of the expression profile of immunoregulatory molecules on the surface of PMPs (37). Based on this work and other published studies, the interaction of MPs with cells and the modulations of their proteins and genes are known to be closely linked to the numbers and phenotype of MPs (27, 28, 30, 33, 34, 38, 39). We therefore hypothesized that the increase in MP numbers in PLWH may lead to phenotypic and functional changes in the immune system potentially associated with chronic immune activation.

We investigated the functional impact on immune system cells of plasma MPs from patients with chronic immune activation, using PBMCs from healthy donors without HIV (HDs). Indeed, it was not possible to use autologous cells for this study because the cells of the patients are already affected by their own MPs. We therefore investigated rapid interactions of MPs from PLWH with CD4^+^ and CD8^+^ TLs, monocytes and DCs, and assessed the phenotypic and functional changes in these cells. We also performed the same experiments with MPs from HDs as a control.

We developed and used an original technique for this purpose in which the cells and MPs are separately and specifically labeled with antibodies targeting proteins of interest conjugated to different fluorochromes (30–32, 34, 38). We focused on three types of MPs, of different cellular origins, known to have significant immunoregulatory potentials (27, 28, 30, 33, 34, 37, 38, 40): MPs from platelets (CD41a^+^ PMPs), monocytes (CD14^+^ MMPs) and T lymphocytes (CD3^+^ LMPs). We mimicked the increase in MP levels in PLWH reported in previous studies (37) by establishing cocultures of different ratios of PBMCs and MPs. Following the interaction of immune cells and MPs, we performed functional studies in which we assessed the secretion of six cytokines (IFNγ, IL2, IL6, IL12, IL17 and TNFα). These cytokines were selected for study because their involvement in cell-MP interactions and immunoregulation has already been described (27–29, 33, 34). The polyfunctionality of secretory cells and the origin of the MPs involved in these functional modifications were then assessed.

The results obtained confirm our hypotheses concerning the importance and functional role of MPs, and of MMPs and PMPs in particular. They also confirm that functional remodeling occurs during interactions with cells of the immune system.

## Methods

### Ethics statement

The study was approved by the local ethics committee, the Comité de Protection des Personnes – Ile de France IX (CPP n°10-023) of Centre Hospitalier Universitaire Henri-Mondor. All participants gave written informed consent.

### Biological samples

Fresh blood samples from healthy donors (HDs) were provided by the French national blood bank (*Etablissement Français du Sang*, EFS). PBMCs were collected in tubes containing sodium heparin (BD Biosciences, Franklin Lakes, NJ) and isolated by density gradient centrifugation, as previously described (41). None of the HDs had suffered an infection (bacterial, viral, fungal, yeast) or had been vaccinated in the 30 days preceding inclusion.

Fresh blood samples from PLWH were collected in tubes containing acid citrate dextrose solution B (ACD-B) (BD Biosciences). PLWH were followed at the infectious diseases and clinical immunology department of Henri-Mondor University Hospital (Creteil, France). As previously described (37), the patients included had HIV infection with at least five years of follow-up. They were on active antiretroviral therapy, had an undetectable viral load (< 50 copies/mL) and had a CD4/CD8 T-cell ratio greater than 0.5. Demographic, clinical and treatment characteristics were collected, together with virological outcomes (**Supplementary Tables 1**, **2**). The study was approved by the local ethics committee, and all participants gave written informed consent.

### MP-enriched EV preparation

EVs were isolated as previously described (26, 30–32, 37, 38, 42). Briefly, the EV was enriched in MPs by differential centrifugation of blood from PLWH or HDs at an initial speed of 3,000 x *g* at 4°C for 10 minutes. The supernatant was then centrifuged at 13,000 x *g* at 4°C for 10 minutes for the preparation of a platelet-free supernatant. EVs enriched in MPs were then concentrated by centrifuging the platelet-free supernatant for 1 hour at 100,000 x *g* at 4°C. The resulting pellet, corresponding to “MP-enriched EVs”, was resuspended in filter-sterilized PBS (filtration through a PES membrane with 0.1 μm pores) and MPs were characterized by flow cytometry.

### MP labeling and counting

MPs from the preparation of MP-enriched EVs were labeled with fluorochrome-conjugated monoclonal antibodies, as previously described (31, 32, 37, 38, 42). MPs were labeled by incubation with three different antibodies (anti-CD41a, anti-CD14, anti-CD3 antibodies) described in **Supplementary Table 3**. Fluorescence was assessed with a 20-parameter LSR Fortessa flow cytometer with a small-particle option (BD Biosciences) based on photomultiplier (PMT)-coupled forward scatter (FSC) detection. This mode of detection was used to ensure the optimal detection of MPs with diameters of 200 to 900 nm. The performance of the flow cytometer was checked before each assay. Megamix-Plus FSC and SSC beads (BioCytex, Marseille, France) of known dimensions (beads with diameters ranging from 200 nm to 900 nm: 200 nm, 240 nm, 300 nm, 500 nm and 900 nm, (**Supplementary Figure 1**) were used for the standardization of FSC-PMT parameters and definition of the MP gate.

MPs were acquired at low speed and quantified in Trucount tubes (BD Biosciences), as previously described (31, 32, 37, 38, 42). Trucount tubes contain a known number of beads. The absolute number of labeled MPs in each sample was determined with the following formula: (number of MPs counted x number of Trucount beads in the tube)/number of Trucount beads counted.

### Interaction of MPs with immune cells and cytokine secretion

The cocultures were performed with MPs derived from the blood of patients (PLWH, *n*=22) or healthy donors (HD, *n*=10), with PBMC from HD (*n=*15) (10 coculture experiments for PLWH and 2 coculture experiments for HD). To maintain experimental conditions as close as possible to *in vivo* environment, MPs were not purified prior to use. Co-cultures were performed using the total bulk of MPs, in which the specific subpopulations of interest (LMPs, MMPs, and PMPs) were labeled with fluorochrome-conjugated antibodies (anti-CD3, anti-CD14, and anti-CD41a, respectively). After labeling, a washing step was performed to remove any unbound antibodies before adding the MPs to the PBMC cultures. The PBMC: MPs ratio was calculated based only on the labeled MPs of interest.

PBMCs were cultured for 18 hours at a density of 1 x 10^5^ cells with known numbers of CD41a-expressing MPs + CD14-expressing MPs + CD3-expressing MPs. The MPs were added to the culture at ratios of 1:1 or 1:20 (PBMCs: MPs) in filter-sterilized (passage through a filter with 0.1 µm pores) culture medium. As a control, cells were also cultured without MPs. The culture medium consisted of RPMI 1640 supplemented with 5% FBS (Dutscher, Bernolsheim, France), 2 mM L-glutamine, 100 µg/ml penicillin/streptomycin, MEM non-essential amino acids solution (1X), and 1 mM sodium pyruvate (all from Thermo Fisher Scientific, Waltham, MA).

For studies of the interactions of MPs expressing CD41a, CD14 or CD3 with PBMCs, cells were harvested after coculture and stained with the membrane marker antibodies described in **Supplementary Table 3** to evaluate the co-expression of the MP markers (labeled with anti-CD41a or anti-CD14 or anti-CD3 antibodies during MP labeling) by flow cytometry. Cells interacting with EVs were collected by flow cytometry, with a minimum of 10,000 cells collected for each cell subpopulation.

The functional impact of MPs expressing CD41a, CD14, or CD3 on cytokine secretion by PBMCs was assessed by fixing and permeabilizing the cells with the Fix & Perm kit (Thermo Fisher Scientific) according to the manufacturer’s instructions. Intracellular markers were detected with the antibodies described in **Supplementary Table 3**. Cytokine secretion by immune cells was assessed by flow cytometry (**Supplementary Figure 2**).

### Flow cytometry analysis

For MP counting, interaction and functional assays, fluorescence was assessed on an LSR Fortessa flow cytometer (BD Biosciences). The flow cytometry data were analyzed with FlowJo software (v.10.7.1, FlowJo, Ashland, OR).

### Statistical analysis

All analyses were performed with Prism 6.07 software (GraphPad Software, La Jolla, CA). Only significant differences between groups (*P*<0.05) are indicated on the data plots. Details of the statistical tests performed are provided in the figure legends.

## Results

### Interactions of CD41a^+^ PMPs, CD14^+^ MMPs and CD3^+^ LMPs with immune cells

**Figure 1:**
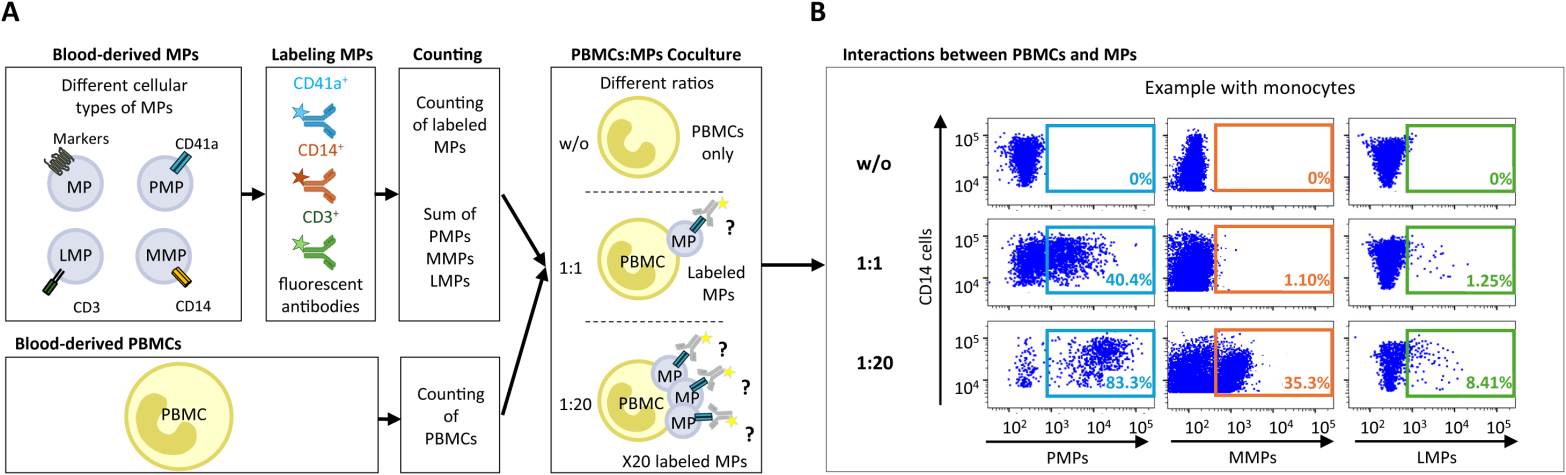
Assays of MP coculture with PBMCs to study interactions between MPs and immune cells. **(A)** Schematic representation of MP coculture assays with PBMCs at different ratios for investigations of the interaction of CD41a^+^ PMPs, CD14^+^ MMPs and CD3^+^ LMPs with immune cells. MPs were isolated and labeled with an anti-CD41a antibody for PMPs, an anti-CD14 antibody for MMPs and an anti-CD3 antibody for LMPs. The total number of labeled MPs (CD41a^+^ PMPs + CD14^+^ MMPs + CD3^+^ LMPs) was calculated by flow cytometry with Trucount beads. The MPs were then cocultured with PBMCs at ratios of 1:1 and 1:20 (PBMCs: MPs). As a control, PBMCs were cultured without MPs (w/o). After coculture, cells that had and had not interacted with MPs were harvested and labeled to study the co-expression of cell markers with CD41a from PMPs, CD14 from MMPs or CD3 from LMPs. **(B)** Example of the gating strategy used for flow cytometric assessment of the interaction of CD41a^+^ PMPs, CD14^+^ MMPs and CD3^+^ LMPs with monocytes (CD14^+^) for one representative experiment.

PBMCs from HDs were cultured with CD41a^+^ PMPs, CD14^+^ MMPs and CD3^+^ LMPs from PLWH or blood-derived MPs from HDs as a control, to investigate the interactions of PLWH MPs with the following immune cells: CD4^+^ TLs, CD8^+^ TLs, DCs (CD11c^+^/CD123^+^ cells), and monocytes (CD14^+^ cells) (**Figure 1A**). The cultures were set up with different ratios of PBMCs to MPs (PBMCs: MPs), to mimic the variation of MP levels in PLWH while maintaining the proportions of the different MP populations, as previously described (37). These interactions were then assessed by flow cytometry, with PMPs, MMPs and LMPs labeled with anti-CD41a APC-H7, anti-CD14 BUV395 and anti-CD3 BV711 antibodies, respectively, and the co-expression of MP markers on the surface of cells was evaluated (**Figure 1B**).

**Figure 2:**
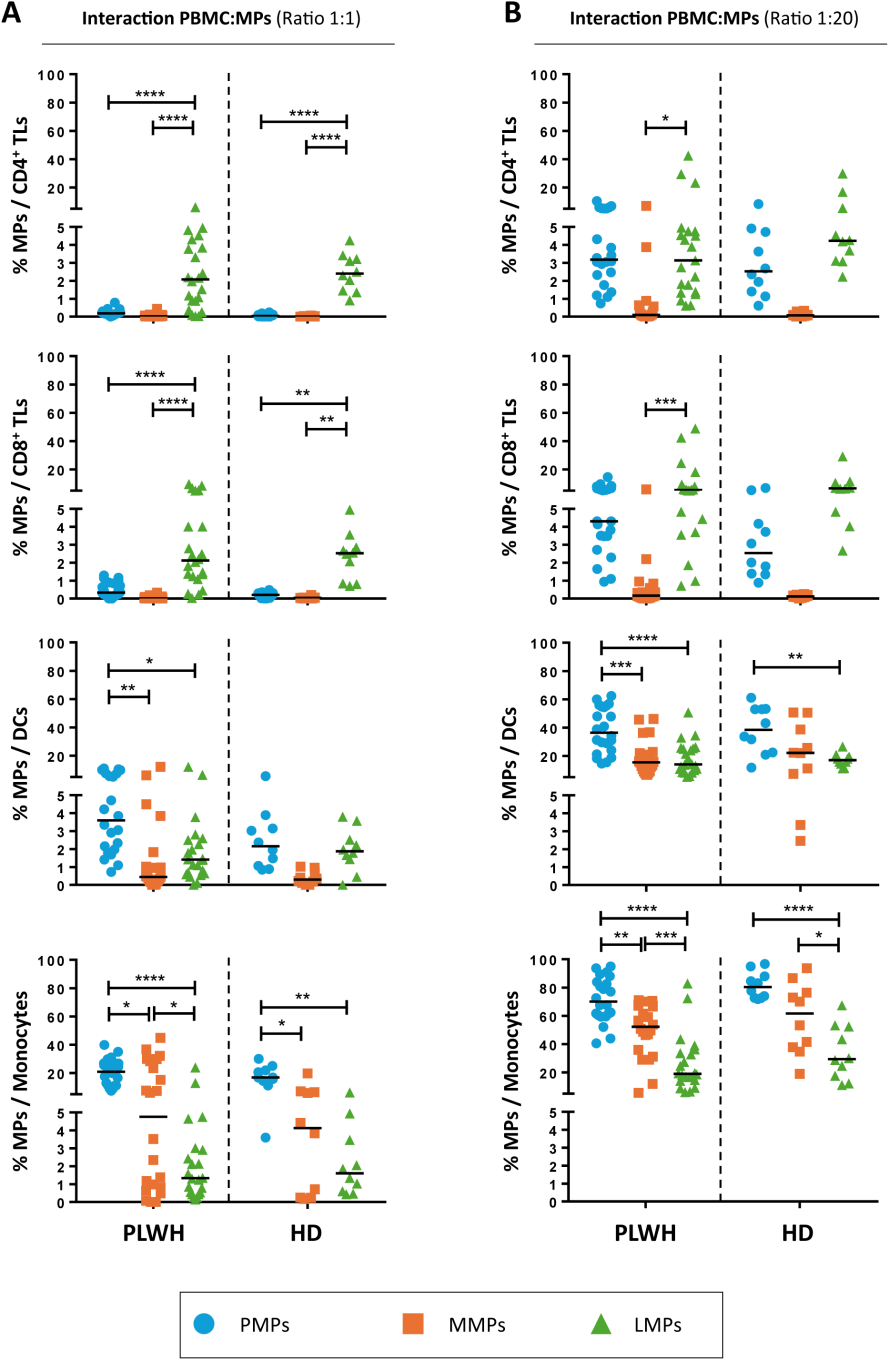
Interaction of CD41a^+^ PMPs, CD14^+^ MMPs and CD3^+^ LMPs with immune cells. **(A, B)** Percentage of cells expressing CD41a from PMPs (blue circle), CD14 from MMPs (orange square) or CD3 from LMPs (green triangle) of patients (PLWH, *n=*22) or healthy donors (HD, *n*=10) at coculture ratios of 1:1 **(A)** or 1:20 **(B)** (PBMCs: MPs). Horizontal bars indicate the median value. *P* values (*P*<0.05 considered significant) were obtained in ANOVA and Kruskal-Wallis *post hoc* tests. ****P<0.0001, ***P<0.001, **P<0.01, *P<0.05.

TLs were the cells that interacted least with the studied MPs. Whatever the culture ratio, CD3^+^ LMPs were the PLWH EVs that interacted the most with TLs, at mean rates of 2.3 ± 0.4% with CD4^+^ TLs and 2.9 ± 0.6% with CD8^+^ TLs at a ratio of 1:1 (PBMCs: MPs) (**Figure 2A**). The rate of interaction between CD3^+^ LMPs and TLs was even greater for a ratio of 1:20 (PBMCs: MPs), at 6.9 ± 2.4% for CD4^+^ TLs and 10.5 ± 3.0% for CD8^+^ TLs (**Figure 2B**). However, even at the maximum ratio, no more than 5% of TLs interacted with CD41a^+^ PMPs and less than 1% interacted with CD14^+^ MMPs (**Figure 2B**). The results obtained with MPs from HDs were similar to those obtained with MPs from PLWH.

CD14^+^ MMPs and CD41a^+^ PMPs interacted only weakly with TLs, instead interacting predominantly with DCs and monocytes. Indeed, at the 1:1 ratio (PBMCs: MPs), CD14^+^ MMPs from PLWH interacted with a mean of 1.6 ± 0.6% of DCs and 12.4 ± 3.1% of monocytes, whereas CD41a^+^ PMPs from PLWH interacted significantly more frequently with these cells, with interaction rates of 4.7 ± 0.7% for DCs and 20.8 ± 1.9% for monocytes (**Figure 2A**). Similarly, for the 1:20 ratio (PBMCs: MPs), DCs bound fewer CD14^+^ MMPs and CD41a^+^ PMPs from PLWH than monocytes, with 18.9 ± 2.6% and 38.33 ± 3.4% of cells, respectively, presenting interactions (**Figure 2B**). Monocytes were the principal cells binding CD14^+^ MMPs and CD41a^+^ PMPs from PLWH, with binding observed to 49.9 ± 4.1% and 71.34 ± 3.4%, respectively, of CD14^+^ cells (**Figure 2B**). Once again, similar results were obtained with MPs from HDs with no significant difference between MPs from PLWH and HDs (**Figure 2**).

### Functional impact of CD41a^+^ PMPs, CD14^+^ MMPs and CD3^+^ LMPs on immune cells

**Figure 3:**
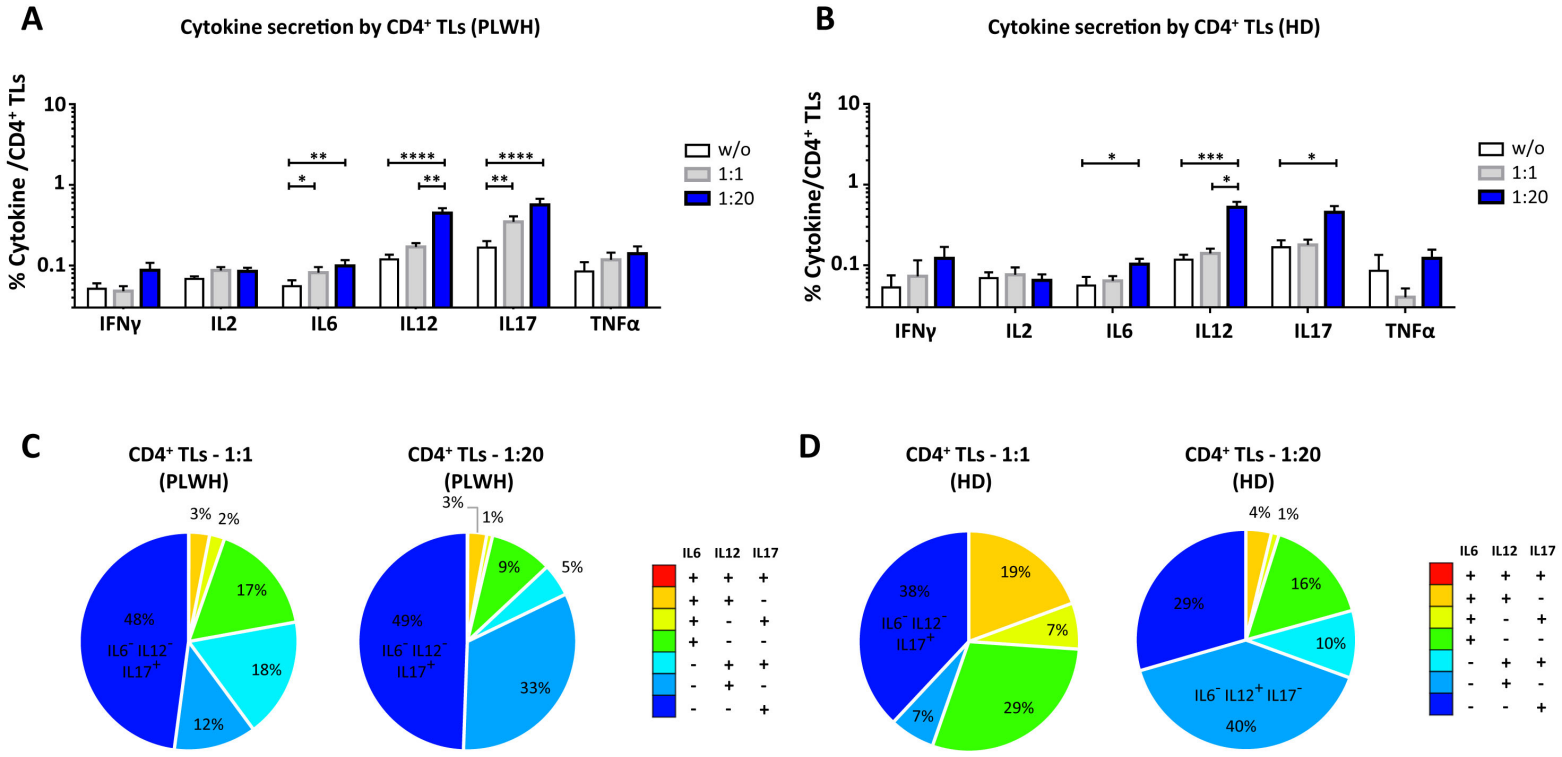
Functional impact of MPs on cytokine secretion by CD4^+^ T lymphocytes. PBMCs were cocultured for 18 hours with known numbers of CD41a^+^ PMPs and CD14^+^ MMPs and CD3^+^ LMPs at ratios of 1:1 and 1:20 (PBMCs: MPs). As a control, PBMCs were also cultured without MPs (w/o). The cells were then harvested, fixed, permeabilized and labeled to investigate cytokine secretion. **(A, B)** The percentages (mean ± SD) of CD4^+^ T lymphocytes secreting IFNγ, IL2, IL6, IL12, IL17 and TNFα were determined without (white bars) or with coculture with MPs from PLWH [**(A)**, *n*=22, except for TNF, *n*=16, 10 independent coculture experiments] or HDs [**(B)**, *n=*10, except for TNF *n*=6, three independent coculture experiments] at ratios of 1:1 (gray bars) and 1:20 (blue bars) (PBMCs: MPs). **(C, D)** Co-expression data for IL6, IL12 and IL17 are presented for CD4^+^ T lymphocytes cocultured with MPs from PLWH [**(C)**, *n*=16, eight independent coculture experiments] or HDs [**(D)**, *n*=6, two independent coculture experiments] at ratios of 1:1 and 1:20 (PBMCs: MPs). *P* values (*P*<0.05 considered significant) were obtained in ANOVA and Friedman’s *post hoc* tests. ****P<0.0001, ***P<0.001, **P<0.01, *P<0.05.

**Figure 4:**
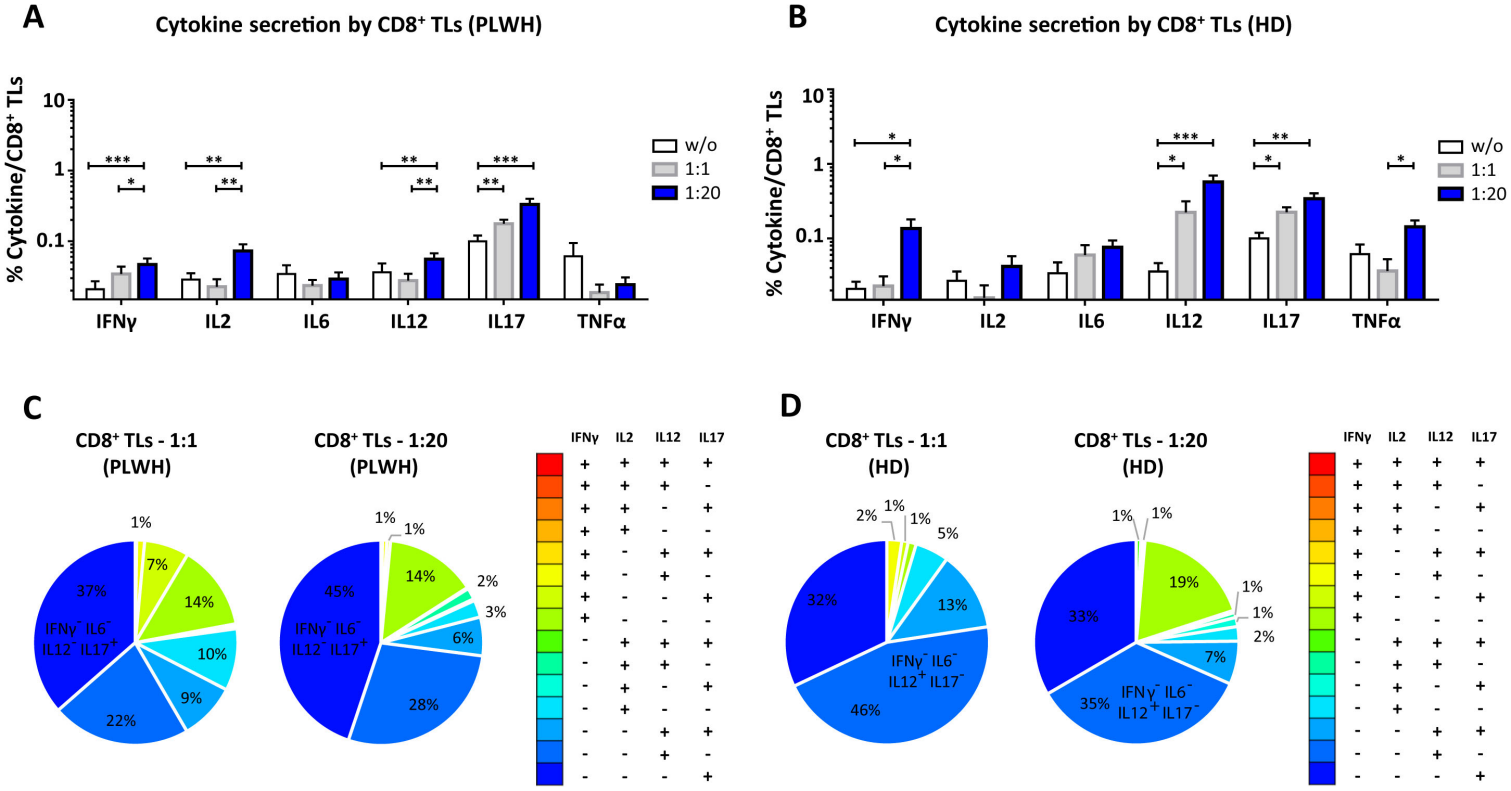
Functional impact of MPs on cytokine secretion by CD8^+^ T lymphocytes. PBMCs were cocultured for 18 hours with known numbers of CD41a^+^ PMPs and CD14^+^ MMPs and CD3^+^ LMPs at ratios of 1:1 and 1:20 (PBMCs: MPs). As a control, PBMCs were also cultured without MPs (w/o). The cells were then harvested, fixed, permeabilized and labeled to investigate cytokine secretion. **(A, B)** The percentages (mean ± SD) of CD8^+^ T lymphocytes secreting IFNγ, IL2, IL6, IL12, IL17 and TNFα were determined without (white bars) or with coculture with MPs from PLWH (**(A)**, *n*=22, except for TNF, *n*=16, 10 independent coculture experiments) or HDs [**(B)**, *n=*10, except for TNF *n*=6, three independent coculture experiments] at ratios of 1:1 (gray bars) and 1:20 (blue bars) (PBMCs: MPs). **(C, D)** Co-expression data for IFNγ, IL2, IL12 and IL17 are presented for CD8^+^ T lymphocytes cocultured with MPs from PLWH [**(C)**, *n*=16, eight independent coculture experiments] or HDs [**(D)**, *n*=6, two independent coculture experiments] at ratios of 1:1 and 1:20 (PBMCs: MPs). *P* values (*P*<0.05 considered significant) were obtained in ANOVA and Friedman’s *post hoc* tests. ***P<0.001, **P<0.01, *P<0.05.

**Figure 5:**
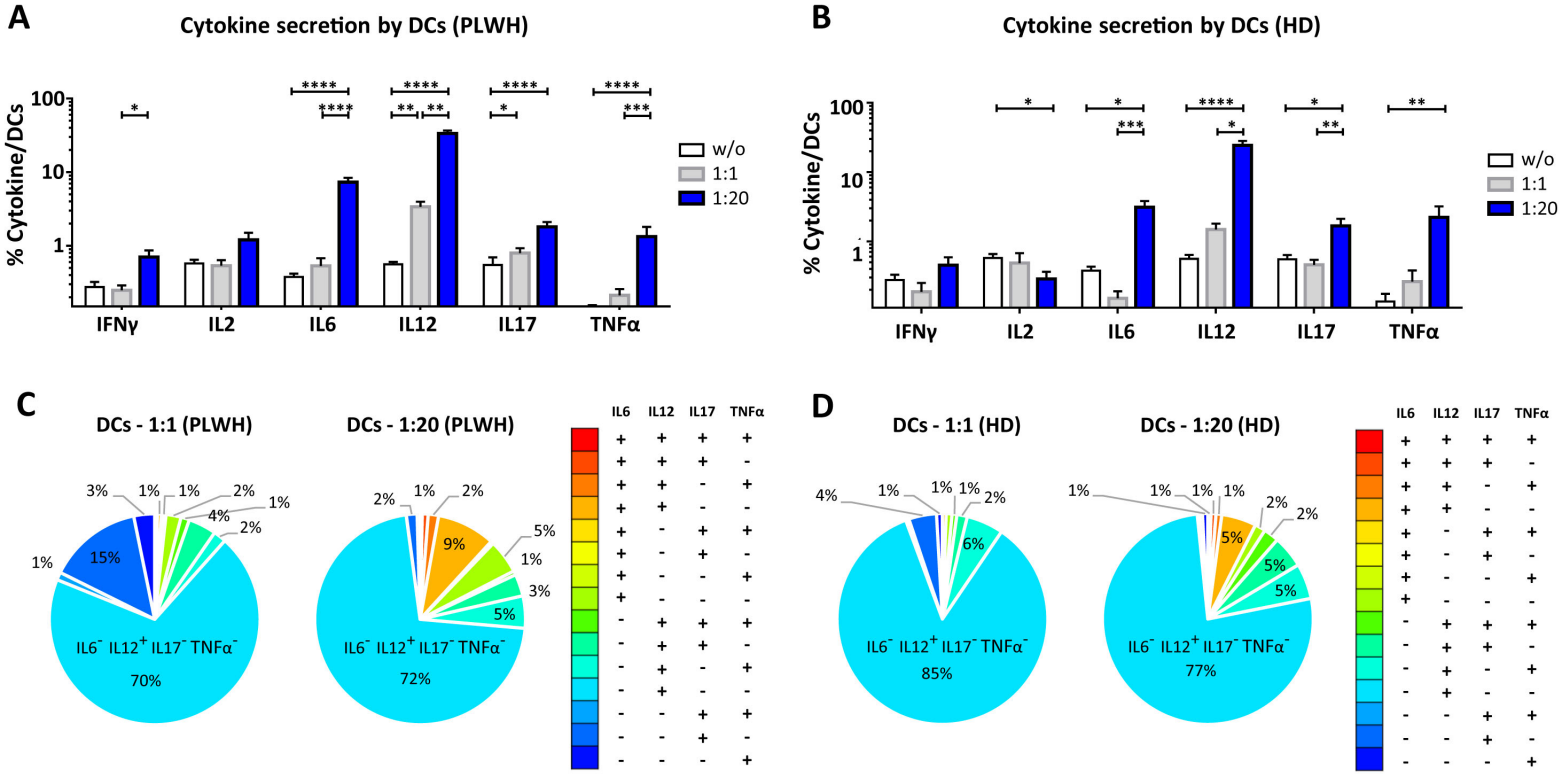
Functional impact of MPs on cytokine secretion by DCs. PBMCs were cocultured for 18 hours with known numbers of CD41a^+^ PMPs and CD14^+^ MMPs and CD3^+^ LMPs at ratios of 1:1 and 1:20 (PBMCs: MPs). As a control, PBMCs were also cultured without MPs (w/o). The cells were then harvested, fixed, permeabilized and labeled to investigate cytokine secretion. **(A, B)** The percentages (mean ± SD) of DCs secreting IFNγ, IL2, IL6, IL12, IL17 and TNFα were determined without (white bars) or with coculture with MPs from PLWH [**(A)**, *n*=22, except for TNF, *n*=16, 10 independent coculture experiments] or HDs [**(B)**, *n=*10, except for TNF *n*=6, three independent coculture experiments] at ratios of 1:1 (gray bars) and 1:20 (blue bars) (PBMCs: MPs). **(C, D)** Co-expression data for IL6, IL12, IL17 and TNFα are presented for DCs cocultured with MPs from PLWH [**(C)**, *n*=16, eight independent coculture experiments] or HDs [**(D)**, *n*=6, two independent coculture experiments] at ratios of 1:1 and 1:20 (PBMCs: MPs). *P* values (*P*<0.05 considered significant) were obtained in ANOVA and Friedman’s *post hoc* tests. ****P<0.0001, ***P<0.001, **P<0.01, *P<0.05.

**Figure 6:**
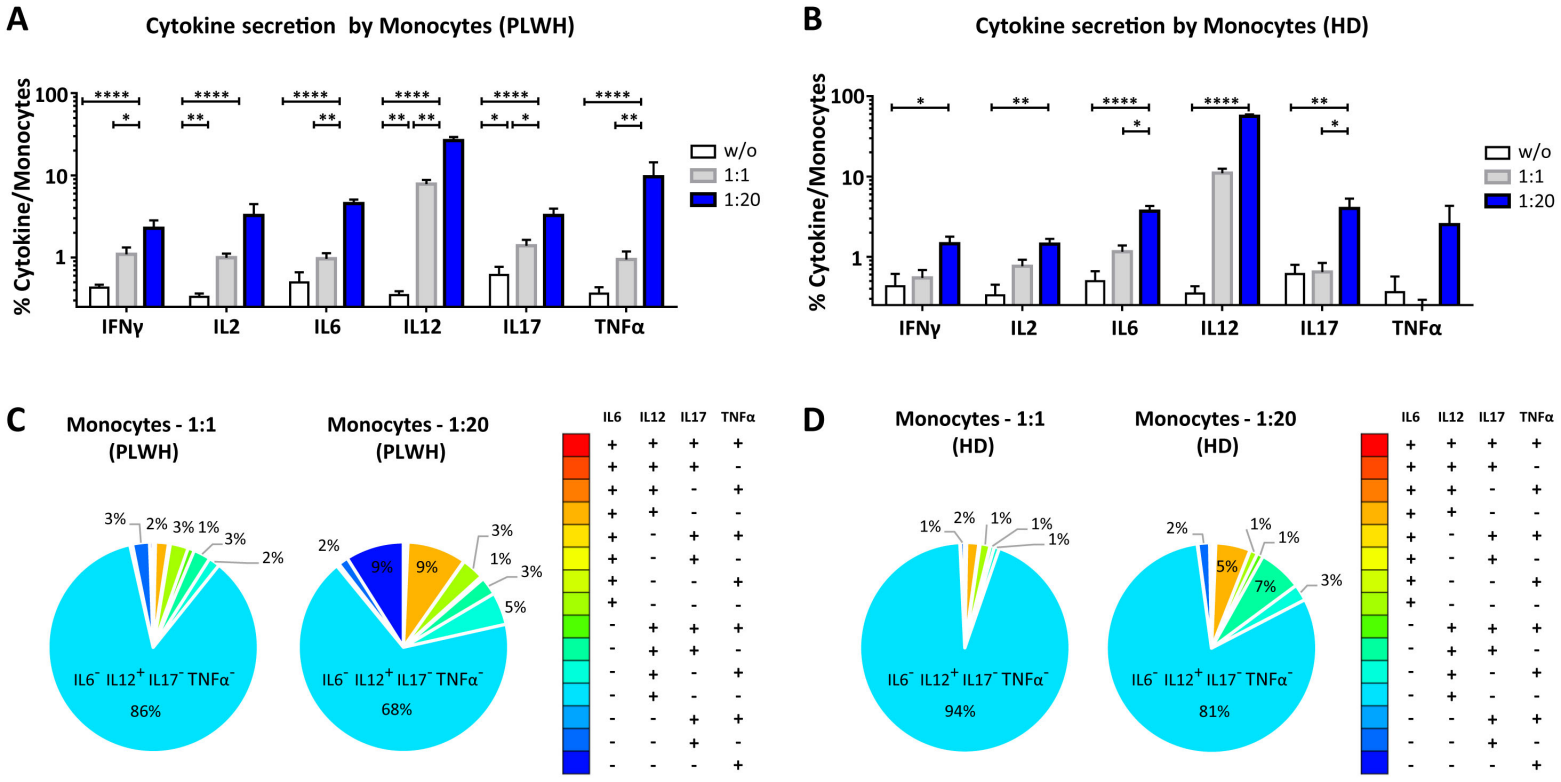
Functional impact of MPs on cytokine secretion by monocytes. PBMCs were cocultured for 18 hours with known numbers of CD41a^+^ PMPs and CD14^+^ MMPs and CD3^+^ LMPs at ratios of 1:1 and 1:20 (PBMCs: MPs). As a control, PBMCs were also cultured without MPs (w/o). The cells were then harvested, fixed, permeabilized and labeled to investigate cytokine secretion. **(A, B)** The percentages (mean ± SD) of monocytes secreting IFNγ, IL2, IL6, IL12, IL17 and TNFα were determined without (white bars) or with coculture with MPs from PLWH [**(A)**, *n*=22, except for TNF, *n*=16, 10 independent coculture experiments] or HDs [**(B)**, *n=*10, except for TNF *n*=6, three independent coculture experiments] at ratios of 1:1 (gray bars) and 1:20 (blue bars) (PBMCs: MPs). **(C, D)** Co-expression data for IL6, IL12, IL17 and TNFα are presented for monocytes cocultured with MPs from PLWH [**(C)**, *n*=16, eight independent coculture experiments] or HDs [**(D)**, *n*=6, two independent coculture experiments] at ratios of 1:1 and 1:20 (PBMCs: MPs). *P* values (*P*<0.05 considered significant) were obtained in ANOVA and Friedman’s *post hoc* tests. ****P<0.0001, **P<0.01, *P<0.05.

**Figure 7:**
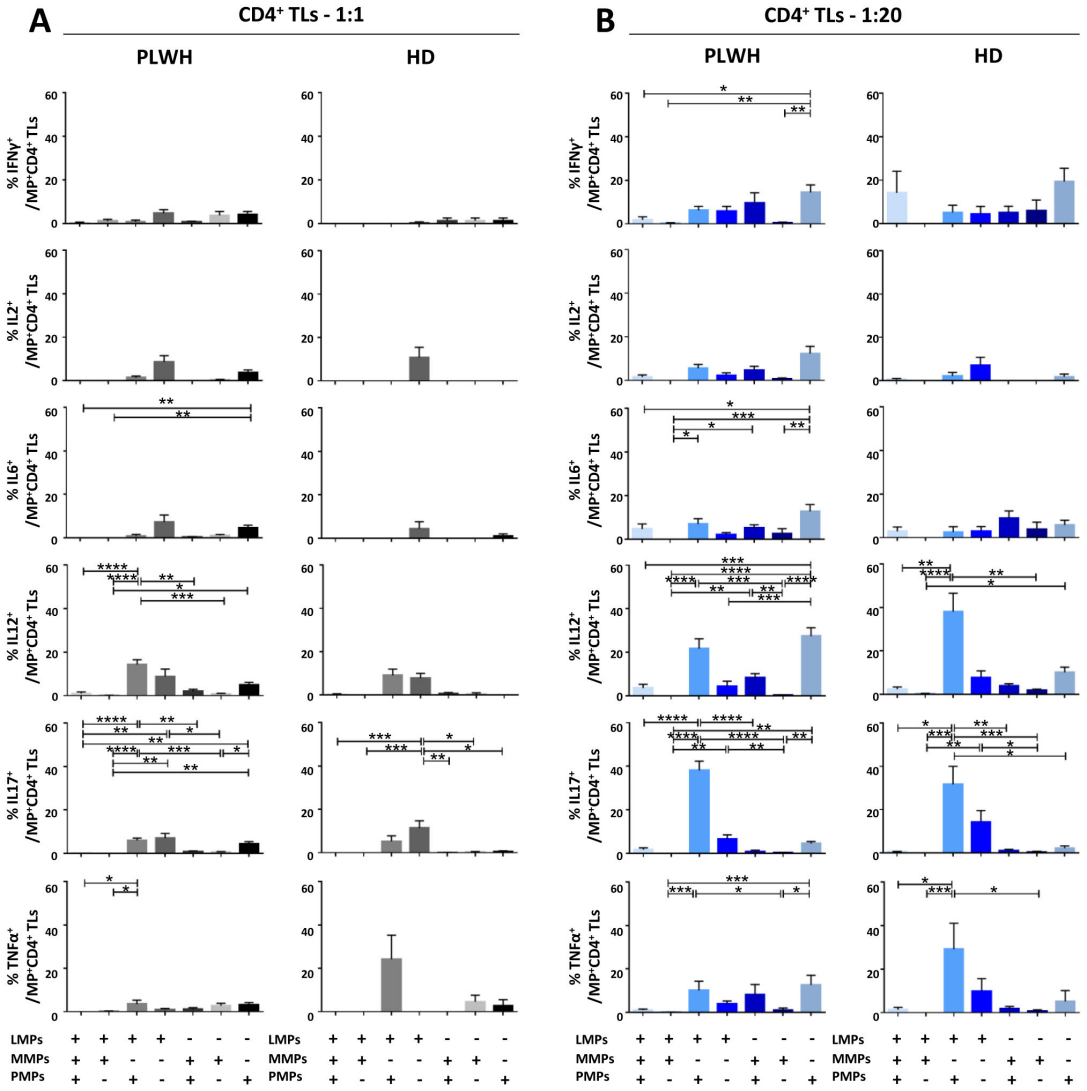
Identification of the MP subpopulations involved in cytokine secretion by CD4^+^ T lymphocytes. **(A, B)** Co-expression data for CD41a^+^ PMPs, CD14^+^ MMPs and CD3^+^ LMPs are presented for each cytokine secreted by CD4^+^ T lymphocytes cocultured with MPs from PLWH (*n*=22, except for TNF, *n*=16, 10 independent coculture experiments) or HDs (*n=*10, except for TNF *n*=6, three independent coculture experiments) at ratios of 1:1 **(A)** or 1:20 **(B)** (PBMCs: MPs). *P* values (*P*<0.05 considered significant) were obtained in ANOVA and Friedman’s *post hoc* tests. ****P<0.0001, ***P<0.001, **P<0.01, *P<0.05.

**Figure 8:**
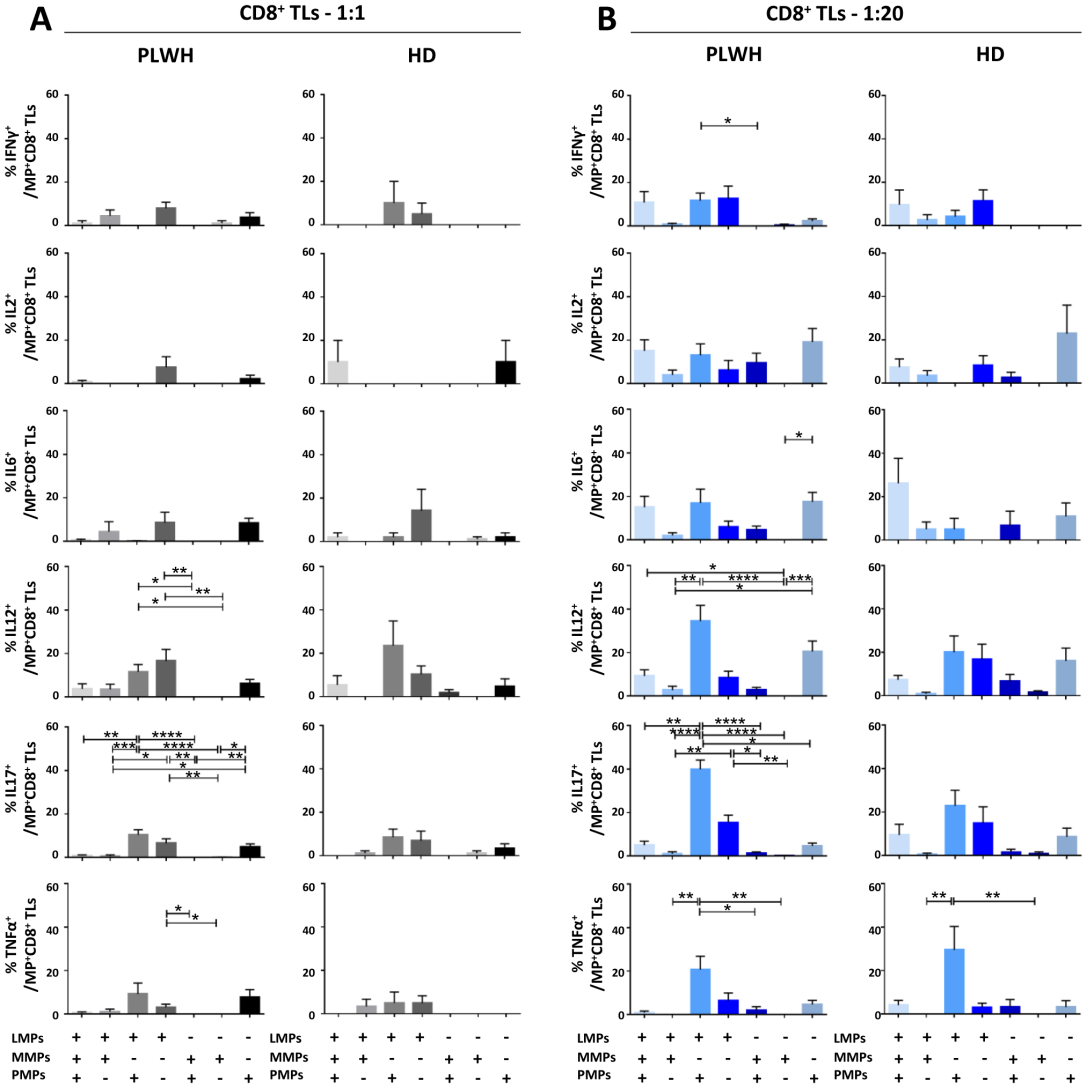
Identification of the MP subpopulations involved in cytokine secretion by CD8^+^ T lymphocytes. **(A, B)** Co-expression data for CD41a^+^ PMPs, CD14^+^ MMPs and CD3^+^ LMPs are presented for each cytokine secreted by CD8^+^ T lymphocytes cocultured with MPs from PLWH (*n*=22, except for TNF, *n*=16, 10 independent coculture experiments) or HDs (*n=*10, except for TNF *n*=6, three independent coculture experiments) at ratios of 1:1 **(A)** or 1:20 **(B)** (PBMCs: MPs). *P* values (*P*<0.05 considered significant) were obtained in ANOVA and Friedman’s *post hoc* tests. ****P<0.0001, ***P<0.001, **P<0.01, *P<0.05.

**Figure 9:**
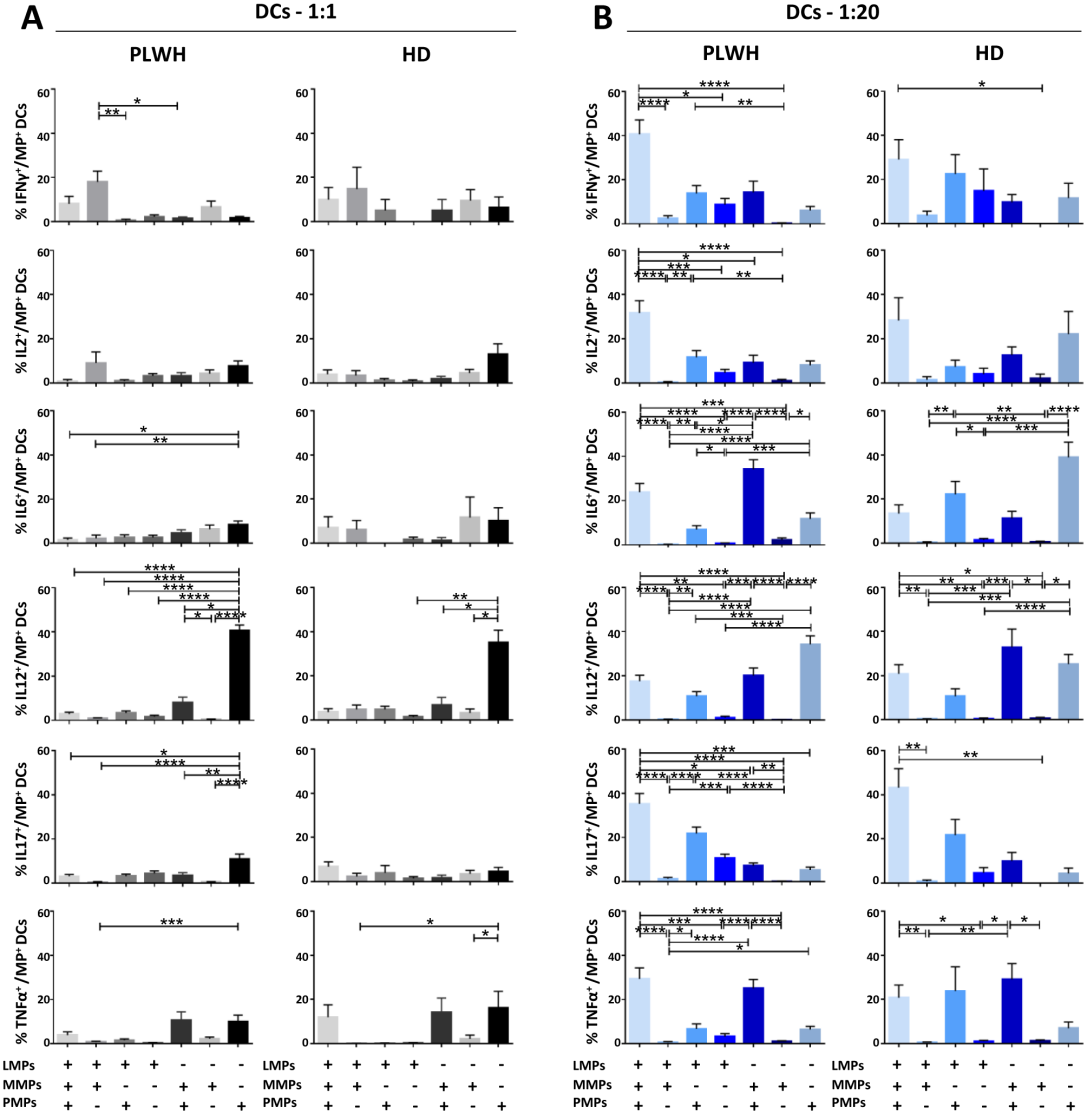
Identification of the MP subpopulations involved in cytokine secretion by DCs. **(A, B)** Co-expression data for CD41a^+^ PMPs, CD14^+^ MMPs and CD3^+^ LMPs are presented for each cytokine secreted by DCs cocultured with MPs from PLWH (*n*=22, except for TNF, *n*=16, 10 independent coculture experiments) or HDs (*n=*10, except for TNF *n*=6, three independent coculture experiments) at ratios of 1:1 **(A)** or 1:20 **(B)** (PBMCs: MPs). *P* values (*P*<0.05 considered significant) were obtained in ANOVA and Friedman’s *post hoc* tests. ****P<0.0001, ***P<0.001, **P<0.01, *P<0.05.

**Figure 10:**
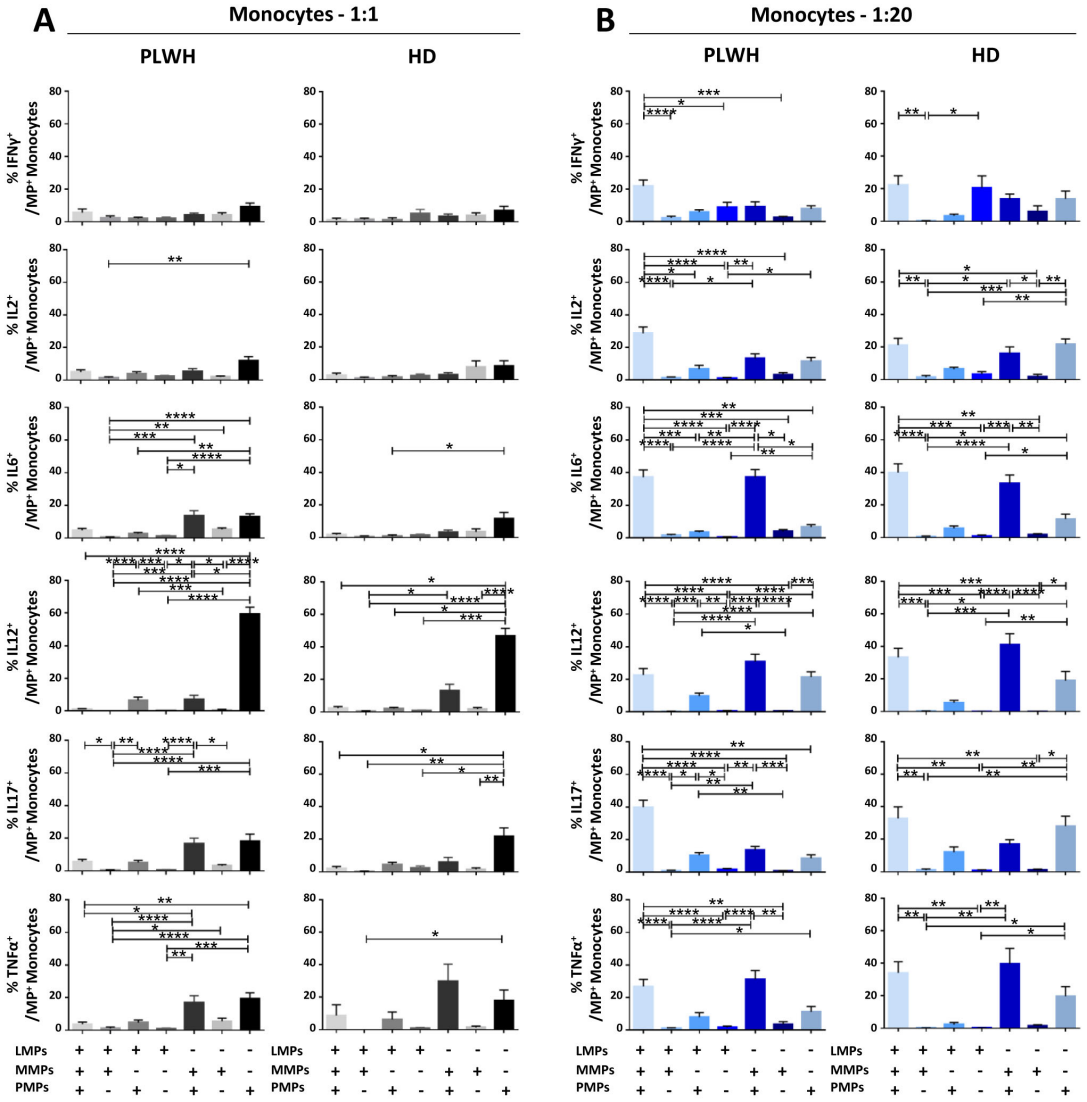
Identification of the MP subpopulations involved in cytokine secretion by monocytes. **(A, B)** Co-expression data for CD41a^+^ PMPs, CD14^+^ MMPs and CD3^+^ LMPs are presented for each cytokine secreted by monocytes cocultured with MPs from PLWH (*n*=22, except for TNF, *n*=16, 10 independent coculture experiments) or HDs (*n=*10, except for TNF *n*=6, three independent coculture experiments) at ratios of 1:1 **(A)** or 1:20 **(B)** (PBMCs: MPs). *P* values (*P*<0.05 considered significant) were obtained in ANOVA and Friedman’s *post hoc* tests. ****P<0.0001, ***P<0.001, **P<0.01, *P<0.05.

After studying the interaction of PLWH MPs or HDs MPs with immune cells, we investigated the phenotypic and functional impact of MPs on these cells. We assessed the secretion of IFNγ, IL2, IL6, IL12, IL17 and TNFα by CD4^+^ TLs, CD8^+^ TLs, DCs and monocytes that had or had not interacted with the MPs studied (**Figures 3A, B**, **4A, B**, **5A, B**, **6A, B**). We recently demonstrated that the number of MPs is significantly higher in PLWH than in healthy controls (37). We therefore performed PBMC: MPs cocultures at different ratios to mimic this variation. We also investigated whether these MPs were responsible for polyfunctional secretion by studying the cosecretion of the targeted cytokines for each cell type (**Figures 3C, D**, **4C, D**, **5C, D**, **6C, D**). Finally, we aimed to determine the cellular origin of the MPs involved in cytokine secretion by analyzing the MP subpopulations co-expressed by secretory cells (**Figures 7**–**10**). Detailed data are provided below for MPs from PLWH and HDs.

### Functional impact of EVs on CD4^+^ TLs

CD4^+^ TLs are known to play a key role in regulating the immune response. We therefore investigated the functional impact of MPs on cytokine secretion by CD4^+^ T cells (**Figures 3**, **7**). CD4^+^ T cells cocultured with PLWH MPs at a ratio of 1:20 (PBMCs: MPs) secreted significantly more IL6, IL12 and IL17 than CD4^+^ TLs without MPs (0.10 ± 0.02% *vs*. 0.06 ± 0.01% for IL6, 2-fold, *P*<0.01, 0.45 ± 0.07% *vs*. 0.12 ± 0.02% for IL12, 4-fold, *P*<0.0001 and 0.57 ± 0.11% *vs*. 0.17 ± 0.03% for IL17, 3-fold, *P*<0.0001) (**Figure 3A**). By contrast, no significant differences were observed for IFNγ, IL2 and TNFα (**Figure 3A**). The same response profile was obtained for MPs from HDs (**Figure 3B**). Furthermore, CD4^+^ cells cocultured with MPs from PLWH did not display polyfunctional cytokine secretion, as shown by a comparative analysis of cytokine co-expression. This analysis revealed that more than 80% of cytokines were secreted individually, with 9.3% ± 9.2% of the cells IL6^+^ IL12^-^ IL17^-^, 32.7% ± 22.9% IL6^-^ IL12^+^ IL17^-^ and 49.4% ± 23.0% IL6^-^ IL12^-^ IL17^+^ for cocultures at a ratio of 1:20 (PBMCs: MPs) (**Figure 3C**). Also, MPs from HDs induced a secretion profile in which individual CD4^+^ TLs predominantly produced a single cytokine with a majority of IL6^-^ IL12^+^ IL17^-^ at a ratio of 1:20(PBMCs: MPs) (**Figure 3D**).

In terms of the cellular origin of the PLWH MPs involved in cytokine secretion, in cocultures at a 1:1 ratio (PBMCs: MPs), CD3^+^ LMPs and CD41a^+^ PMPs alone and together, in interaction with cells accounted for a large proportion of the observed cytokine secretion, particularly for IL12, secreted by 14.3% ± 2.2% of LMP^+^ PMP^+^ cells, 8.8% ± 3.5% of LMP^+^ cells alone and 4.9% ± 1.2% of PMPs^+^ cells alone (**Figure 7A**). By contrast, very few secretory CD4^+^ TLs were MMP^+^ (**Figure 7**). At a ratio of 1:20, this co-expression with CD3 from LMPs and CD41a from PMPs remained significant, almost doubling for IL12, at 21.7% ± 4.5% and increasing six-fold for IL17 (5.9% ± 1.1% at a ratio of 1:1 and 38.2% ± 4.2% at a ratio of 1:20) (**Figure 7B**). Moreover, for all cytokines, and IL12 in particular, the CD41a^+^ PMP population alone played a greater role at a 1:20 ratio (PBMCs: MPs; 27.45% ± 3.8%) than at a 1:1 ratio (4.9% ± 1.2%) (**Figure 7**). The profile of the HD MPs inducing the secretion of these cytokines was identical to that of the PLWH MPs, but with LMPs and PMPs playing respectively a more important role in IL17 secretion and less important role in IL12 secretion (**Supplementary Figure 4**).

### Functional impact of EVs on CD8^+^ TLs

CD8^+^ TLs play an essential role in the immune system, contributing to the suppression and control of viral infections. We therefore assessed the functional impact of MPs on cytokine secretion by CD8^+^ T cells (**Figures 4**, **8**). CD8^+^ T cells cocultured with PLWH MPs at a ratio of 1:20 (PBMCs: MPs) displayed a significant increase, by at least two-fold, in the secretion of IFNγ, IL2, IL12 and IL17 relative to CD8^+^ LTs without EVs (0.05 ± 0.01% *vs*. 0.02 ± 0.01% for IFNγ, *P*<0.001, 0.07 ± 0.02% *vs*. 0.03 ± 0.01% for IL2, *P*<0.01, 0.06 ± 0.01% *vs*. 0.04 ± 0.01% for IL12, *P*<0.01, and 0.33 ± 0.07% *vs*. 0.10 ± 0.02% for IL17, *P*<0.001) (**Figure 4A**). By contrast, no significant difference was observed for IL6 and TNFα (**Figure 4A**). MPs from HDs yielded a similar response profile, except for TNFα at a 1: 20 ratio (PBMCs: HD MPs) (**Figure 4B**). The percentages of cells secreting several cytokines simultaneously were very low, averaging 17% and 10% at ratios of 1:1 and 1:20 (PBMCs: PLWH MPs), respectively (**Figure 4C**). Indeed, CD8^+^ T cells tended to produce a dominant cytokine, principally IL17, which alone accounted for 36.5% ± 25.8% of the cytokines secreted in cultures at a 1:1 ratio (PBMCs: PLWH MPs) and 44.9% ± 27.6% in cultures at a 1:20 ratio (**Figure 4C**). MPs from HDs induced a cytokine secretion profile in which individual CD8^+^ TLs mostly produced a single cytokine (**Figure 4D**).

We found that two types of PLWH MPs were predominantly responsible for the secretion of these cytokines by CD8^+^ LTs: CD3^+^ LMPs and CD41a^+^ PMPs alone were most frequently implicated in cytokine secretion in cultures at a 1:1 ratio (PBMCs: MPs) (**Figure 8A**). Co-expression from PLWH LMPs and PLWH PMPs increased with the number of MPs, particularly for IL12 (34.5% ± 7.2%) and IL17 (39.9% ± 4.3%) in cocultures at a ratio of 1:20 (PBMCs: MPs) (**Figure 8B**). Again, the profile of the HD MPs inducing the secretion of these cytokines was identical to that of the PLWH MPs (**Supplementary Figure 4**).

### Functional impact of EVs on CD11c/CD123^+^ DCs

Dendritic cells (DCs) are crucial to the immune system, acting as sentinels and antigen-presenting cells. We therefore examined the functional impact of MPs on cytokine secretion by CD11c^+^/CD123^+^ DCs (**Figures 5**, **9**). DCs cocultured with PLWH MPs at a ratio of 1:1 (PBMCs: MPs) produced significantly more IL12 than DCs cultured in control conditions (without MPs) (3.39 ± 0.59% *vs*. 0.56 ± 0.05%, 6-fold increase, *P*<0.01) (**Figure 5A**). Moreover, for PLWH MPs, the amounts of IL12 secreted were 10 times higher for a culture ratio of 1:20 (33.58 ± 3.15%, *P*<0.01) and 60 times higher than the control (*P*<0.0001) (**Figure 5A**). Furthermore, the amounts of IFNγ (0.71 ± 0.16% *vs*. 0.28 ± 0.05%, 2.5-fold, *P*<0.05), IL6 (7.32 ± 1.05% *vs*. 0.38 ± 0.04%, 3-fold, *P*<0.0001), IL17 (1.81 ± 0. 30% *vs*. 0.55 ± 0.15%, 19-fold*, P*<0.0001) and TNFα (1.34 ± 0.47% *vs*. 0.13 ± 0.03%, 10-fold, *P*<0.0001) secreted by DCs were significantly higher in cultures with MPs than in control conditions without MPs (w/o) (**Figure 5A**). A similar response profile was obtained for MPs from HDs, except for IFNγ and IL2 (**Figure 5B**). Given the many cytokines secreted by DCs in coculture with MPs, we investigated whether single DCs secreted several cytokines simultaneously. Our data indicate that for almost all cytokines, DCs secreted a single cytokine when cocultured at a ratio of 1:1 (PBMCs: PLWH MPs), with a strong predominance of IL12-producing DCs (69.5 ± 19.3% IL6^-^ IL12^+^ IL17^-^ TNFα^-^) (**Figure 5C**). Similarly, at a 1: 20 ratio (PBMCs: MPs), fewer than 15% of cells displayed polyfunctional secretion of IL6, IL12, IL17, and TNFα (**Figures 5C, D**).

This monofunctional effect of MPs on DCs predominantly involved CD41a^+^ PMPs for cocultures at a 1:1 ratio (PBMCs: MPs), particularly for IL12 (40.6 ± 2.5% for CD41a^+^ PMPs alone, 7.9 ± 2.6% for CD41a^+^ PMPs + CD14^+^ MMPs, 3.3 ± 0.9% for CD41a^+^ PMPs + CD3^+^ LMPs and 2.8 ± 0.8% for CD41a^+^ PMPs + CD14^+^ MMPs + CD3^+^ LMPs) (**Figure 9A**). A synergistic effect of CD41a^+^ PMPs and CD14^+^ MMPs from PLWH was observed in cocultures at a ratio of 1:20 (PBMCs: MPs), notably for IL6 and TNFα, with an 8-fold (34.2 ± 4.4% *vs*. 4.5 ± 1.6%) and a two-fold (25.1 ± 4.1% *vs*. 10.6 ± 3.8%) increase in the MMP^+^ PMP^+^ population. The same was true for the three types of PLWH MPs, with 16 (23.9 ± 3.9% *vs*. 1.5 ± 0.9%) and 8 (29.4 ± 5.0% *vs*. 3.9 ± 1.5%) times more IL6 and TNFα, respectively, secreted by DCs interacting with LMPs, MMPs and PMPs (**Figure 9B**). Again, the results obtained with HD MPs were similar to those for PLWH MPs, with more important role of LMPs and PMPs for IL6 secretion (**Supplementary Figure 4**).

### Functional impact of EVs on CD14^+^ monocytes

CD14^+^ monocytes play an essential role in the immune system, particularly in phagocytosis and the activation of adaptive immune responses. We therefore investigated the functional impact of MPs on the secretion of IFNγ, IL2, IL6, IL12, IL17 and TNFα by CD14^+^ monocytes cultured alone or in the presence of EVs at different ratios (PBMCs: MPs) (**Figures 6**, **10**). When CD14^+^ monocytes were stimulated by large numbers of PLWH MPs (ratio 1:20), they had a strong cytokine activation profile, with significantly higher levels of secretion for all the cytokines studied than observed for control monocytes without MPs (w/o) (2. 27 ± 0.55% *vs*. 0.43 ± 0.04% for IFNγ, 3.25 ± 1.23% *vs*. 0.33 ± 0.03% for IL2, 4.54 ± 0.53% *vs*. 0.50 ± 0.17% for IL6, 26.45 ± 2.70% *vs*. 0. 35 ± 0.04% for IL12, 3.24 ± 0.70% *vs*. 0.61 ± 0.16% for IL17 and 9.61 ± 4.72% *vs*. 0.36 ± 0.07% for TNFα, *P*<0.0001 for all) (**Figure 6A**). A similar response profile was obtained with MPs from HDs (**Figure 6B**). Our analysis of the polyfunctional secretion of the cytokines secreted in the largest amounts (IL6, IL12, IL17 and TNFα) by monocytes indicated that, in cocultures at a ratio of 1:1 (PBMCs: PLWH MPs), 85.7% ± 7.8% of monocytes secreted only IL12 (**Figure 6C**). Furthermore, at a ratio of 1:20, monocytes still mostly secreted IL12 alone (67.6% ± 16. 9%) but some simultaneous secretion of IL6, IL17 and TNFα was observed (9.0% ± 5.5% IL6^+^ IL12^+^ IL17^-^ TNFα^-^, 2.8% ± 2.1% IL6^-^ IL12^+^ IL17^+^ TNFα^-^ and 5.0% ± 6.0% IL6^-^ IL12^+^ IL17^-^ TNFα^+^) (**Figure 6C**). Again, a similar response profile was obtained with MPs from HDs (**Figure 6D**).

At a 1:1 ratio (PBMCs: MPs), CD41a^+^ PMPs were the MPs with the greatest effect on monocyte secretion (**Figure 10**). Indeed, a mean of 59.4% ± 4.3% of IL12^+^ monocytes also expressed the CD41a marker of PMPs (**Figure 10A**). At a ratio of 1:20 (PBMCs: MPs), our data testify to a synergistic effect of PMPs with MMPs from PLWH, as about one third of the IL6^+^ (37.2% ± 4.7%), IL12^+^ (30.9% ± 4.6%) and TNFα^+^ (31.3% ± 5.2%) monocytes also carried the markers of PMPs and MMPs. For each of the cytokines studied, at least 20% of monocytes interacted with all three MP types and expressed the markers of PMPs, MMPs and LMPs on their surface (**Figure 10B**). Again, the results obtained with MPs from HD were similar to those obtained with MPs from PLWH (**Supplementary Figure 4**).

## Discussion

We showed in a recent study that the increase in MP levels in PLWH involves MPs of all cell origins except PMPs and MMPs (37). The data presented here suggest that the lack of change in the levels of these two MP populations may be due to a rapid and massive uptake of PMPs and MMPs by immune cells (**Figure 2**). We have shown *in vitro* that PMPs and MMPs bind preferentially to monocytes and dendritic cells, whereas LMPs interact more with T lymphocytes. These preferential interactions may be explained by ligand/receptor binding. Indeed, MPs can express many molecules on their surface, including both markers of cellular origin or coreceptors (32, 34, 37, 38). We have previously shown that CD27^+^ MPs can interact with CD4^+^ TLs via the CD70 receptor and that they bind only to lymphocytes that already express CD27 on their surface (32). These data demonstrate a tropism of MPs, which do not all interact in the same way with immune cells.

We have also shown that MPs from platelet concentrates expressing the CD39 ectoenzyme interact predominantly with monocytes, DCs and B lymphocytes (BLs), and to a lesser extent with TLs. These CD39^+^ MPs co-express other molecules on their surface that may underlie these interactions, such as CD73, CD80, OX40L, TGFβ and BTLA. These data and our recent phenotyping of MPs from platelets, monocytes and lymphocytes therefore support the hypothesis that the preferential interactions of PMPs, MMPs and LMPs may involve the binding of a ligand/receptor pair (34, 37).

Our initial results also confirm that this interaction depends on the numbers of MPs present in addition to their phenotype or cell origin. This notion of the importance of the numbers of MPs has been put forward before (30–32, 34, 38). We used a 1:20 culture ratio (PBMCs: MPs) to mimic the increase in the number of MPs in PLWH (37). We observed that the cells studied — whether monocytes, DCs or lymphocytes — interacted increasingly strongly with MPs as the number of MPs increased (**Supplementary Figure 3**). Importantly, this 1:20 ratio (PBMCs: MPs) was not uniform across all the MP subpopulations present in the mixture of MPs used. For example, for an overall ratio of 1:20 (PBMCs: MPs), PMPs and LMPs were present at a real ratio of 1:13 (PBMCs: PMPs) and 1:4 (PBMCs: LMPs), respectively (**Supplementary Table 4**). Indeed, PMPs accounted for 64.8 ± 8.6% of the MPs studied, whereas MMPs and LMPs accounted for 19.2 ± 5.7% and 16 ± 5.3%, respectively (**Supplementary Figure 1C**). Nevertheless, our study model is representative of the proportions of MPs of different cellular origins present in PLWH (37).

Furthermore, whatever the type of MPs involved, our results show that increasing the number of MPs has a rapid impact on immune cell function. Indeed, the interaction of cells with MPs leads to an *in vitro* rapid increase in cytokine secretion (**Figures 3A, B**, **4A, B**, **5A, B**, **6A, B**). We observed similar increases in cytokine secretion with PMPs purified by flow cytometry (34), reducing the likelihood of another immunoregulatory element being involved. We systematically checked that there was no PAMP contamination. We found that the levels of IL6, IL12 and IL17 secretion by dendritic cells and monocytes and the levels of IL12 and IL17 secretion by TLs increased with the number of MPs. Conversely, the distribution of the cytokines produced varied with the number of cultured MPs (**Figures 3C, D**, **4C, D**, **5C, D**, **6C, D**). For example, at a culture ratio of 1:1 (PBMCs: MPs), about 90% of monocytes secreted IL12 only (IL6^-^IL12^+^IL17^-^TNFα^-^) whereas, at a ratio of 1:20 (PBMCs: MPs), almost 70% of monocytes secreted IL12 only (IL6-IL12+IL17-TNFα-), 10% secreted TNFα only (IL6^-^IL12^-^IL17^-^TNFα^+^) and 10% secreted both IL6 and IL12 (IL6^+^IL12^+^IL17^-^TNFα^-^) (**Figure 6C**).

These cultured MPs are, therefore, far from being able to facilitate a functional TL profile compatible with the control of viral infection, which would involve the secretion of IFNγ, IL2 and TNFα (2, 43, 44). Moreover, in elite controllers, the control of viral infection is characterized by highly efficient and polyfunctional Th1 effector responses (2, 43, 44). By contrast, we found that the MPs of PLWH induced high levels of cytokine production, an ineffective response for the control of chronic viral infections that was neither Th1-like nor polyfunctional.

These single-cytokine secretion profiles, involving the production principally of IL6, IL12 or IL17, are nonetheless of interest for the control of other diseases, such as inflammatory diseases, autoimmune diseases and cancers. These diseases frequently occur as comorbid conditions in HIV-infected patients. The principal modulation observed after interaction with MPs was the massive secretion of IL12 by all cell types, including monocytes. IL12 is a cytokine that plays a key role in immune system activation. It is involved in the induction of IFNγ production by several cell types, including TLs, and underlies the generation of Th1 cells (45, 46). IL12 is usually produced by antigen-presenting cells and phagocytes, but we show here that MPs can induce IL12 secretion not only in DCs and monocytes, but also in TLs. IL12 is known principally for its therapeutic antitumor and anti-infection properties (45, 47, 48). However, it also plays a major role in HIV infection, enabling macrophages to regain their ability to produce IFNγ, thereby improving immune defenses (45, 49). PBMCs from HIV-infected viremic patients have been shown to present a significant immunodeficiency-related defect of IL12 production (50). ART partially restores IL12 production and reduces immune activation (51). Thus, IL12 may play a protective role in the context of HIV infection. However, the MPs of PLWH may trigger and/or maintain IL12 secretion, potentially leading to immune system hyperactivation by promoting pro-inflammatory cytokine production and activating T cells (52–55).

Two other cytokines known to be pro-inflammatory — IL17 and IL6 — are also upregulated in the presence of PLWH MPs. IL17 is produced principally by Th17 cells and promotes the production of chemokines, the main role of which is the promotion of an inflammatory response by attracting immune cells to the infected or injured tissue (56). In addition, IL17 also stimulates the production, by immune cells, of other pro-inflammatory cytokines, such as TNFα and IL6 (56–58). IL6 is produced principally by BLs, TLs and macrophages and promotes the Th2 response by reducing IFNγ production by T cells, leading to an inhibition of Th1 polarization (59–61). Our results show that MPs induce the production of large amounts of IL6 by dendritic cells and monocytes, with very little of this cytokine produced by T cells. Thus, MPs can reorient the *in vitro* functional phenotype of cells by facilitating the secretion of a cytokine by a cell type that could not initially produce the cytokine concerned. Moreover, the induction of IL6 secretion by MPs can counteract the beneficial effects of IL12 by inhibiting the Th1 response. IL6 can also act synergistically with other cytokines to promote the differentiation of CD4^+^ T cells into Th17 cells (59, 62). However, we observed significant IL17 secretion by T cells stimulated with PLWH MPs, suggesting that PLWH MPs can induce the activation of naive T cells and their differentiation into Th17 cells. Our study provides important insights into MP-mediated immune interactions *in vitro. O*f course, these results do not reflect all the complexity of *in vivo* immune responses in patients. Although this limitation, the immune interaction of MPs does exist *in vivo* (30), and should be considered for immuno-monitoring patients.

We found that PLWH MPs induce immune system hyperactivation by promoting the production of pro-inflammatory cytokines, such as IL12, IL17 and IL6. The chronic activation mediated by these cytokines can lead to the development of comorbid conditions, such as autoimmune or chronic inflammatory diseases, and cardiovascular disease (52–55, 59, 63–67). These data suggest that the IL12, IL6 and IL17 secretion induced by PLWH MPs may play a role in the development of certain comorbid conditions in PLWH, such as autoimmune diseases, cancer and cardiovascular diseases.

The heterogeneity of comorbid conditions in PLWH may also be linked to the phenotype of the MPs. Indeed, regardless of the type of cell studied, we found that the functional remodeling of the cells was dependent on the cellular origin of the MPs. For example, IL12 secretion by different cell types was induced by different types of MPs. Indeed, IL12 secretion by T cells depended principally on LMPs and PMPs, acting alone or in synergy (**Figures 7**, **8**), whereas IL12 secretion by monocytes depended principally on PMPs, acting alone or in synergy with MMPs (**Figure 10**). Conversely, MMPs were unable to induce IL12 secretion by immune cells on their own (**Figures 7**–**10**), suggesting that the number and phenotype of MMPs in a PLWH can influence the functional impact of these MPs on immune system cells. The phenotypic heterogeneity of MPs may, thus, be directly linked to the heterogeneity of functional remodeling in the immune system cells studied. This phenotypic heterogeneity of MPs may, therefore, have consequences not only for viral replication, as they do not favor polyfunctional and/or Th1-type responses, but also for the development of comorbid conditions in patients with chronic HIV infection.

One of the limitations of this study is that it did not consider the immunoregulatory profile of the MPs studied. However, we know that the immunoregulatory profile of MPs also influences the functionality of immune cells (30, 32, 38). Indeed, we have previously shown that TGFβ^+^ MPs can activate both Tfh and Treg cells (30). Another study has also shown that CD27^+^ and CD70^+^ MPs can transfer their receptors to CD4^+^ TLs, boosting their activation and lymphoproliferation (32). We recently showed that CD39^+^ MPs have an inhibitory phenotype that can be transferred to immune cells, affecting immunoglobulin production by B cells and the lymphoproliferative capacity of CD4^+^ TLs (38). All these studies demonstrate the importance of explorations going beyond the cellular origin of MPs, as these MPs co-express numerous molecules on their surface that may be involved in interactions, have a functional role, or both. It will, therefore, be important to determine whether functional modifications of immune cells are also observed following the overexpression of immunoregulatory molecules in PLWH (37).

Despite the differences between the immunoregulatory profiles of MPs from PLWH MPs and MPs from HDs (37), their functional impacts were similar (**Figures 3**–**6**). Only the MP subpopulations involved in these changes in immune cell phenotype appeared to differ between HDs and PLWHs, highlighting the importance of studying the complex phenotype of MPs (**Figures 7**–**10**; **Supplementary Figure 4**). However, there is no physiological reason to study cocultures at a ratio of 1:20 (PBMCs: MPs) for MPs from healthy donors. Indeed, the only known difference between the MPs of healthy donors and PLWH concerns the number of MPs, resulting in a much stronger immunoregulatory profile in PLWH (37). The reason for the increase in budding underlying MP production remains unknown — an external source, such as the virus itself or antiretroviral treatments may be involved — but it would be interesting to determine the functional impact on immune cells of limiting MP formation. Certain drugs — including calpeptin, Rho-kinase pathway inhibitors and cholesterol-lowering drugs, such as methyl-β-cyclodextrin and statins — have been reported to inhibit MP formation (68–70).

The results presented here support the hypothesis that extracellular vesicles, including the PMPs, MMPs and LMPs present in the bloodstream of PLWH, can interact strongly with immune system cells, rapidly modulating and reorienting the phenotype and function of these cells in a monofunctional manner. However, the cellular origin of the MPs is not the only phenotypic factor controlling functional remodeling. The MPs of PLWH also express a large number of immunoregulatory molecules (37).

These results provide new insight into the possible functional remodeling of immune system cells according to the phenotype and number of MPs. The reason for the increase in MP number is unknown, but the inclusion of viremic patients and elite controllers in a new study might help answer this question. At this stage, the mechanism by which MPs induce the remodeling of immune cell functions remains unknown. Studies involving a transfer of genetic material should shed light on this mechanism (18, 71, 72).

Moreover, neither the entire immune system nor all the types of MPs have yet been studied. Our results demonstrate the importance of studying the functional impact of the phenotypic heterogeneity of MPs on immune cells. Studies of the impact of all EVs on the many cellular targets of the immune system are not feasible with conventional flow cytometry techniques. Multi-omics approaches might provide more comprehensive multiparametric results (34).

These data are of potential value for the development of new treatments aiming to decrease the impact of these MPs, regardless of whether they are related to infection. Conversely, for other diseases, these findings may facilitate the maintenance of immune activation, but much remains unknown about MPs.

## Data Availability

The original contributions presented in the study are included in the article/**Supplementary Material**. Further inquiries can be directed to the corresponding author.
