# Supplementary material for: Remodeling of immune system functions by extracellular vesicles
Source: Front Immunol. 2025 Mar 13;16:1549107. doi: 10.3389/fimmu.2025.1549107 (PMC11966064; doi:10.3389/fimmu.2025.1549107)
Supplement: Supplementary file 1 [file DataSheet1.pdf]

Remodeling of immune system functions by extracellular vesicles

Deborah Neyrinck-Leglantier, Marie Tamagne, Raida Ben Rayana, Souganya Many, Marion Klea Pinheiro, Adèle Silane Delorme, Muriel Andrieu, Eric Boilard, Fabrice Cognasse, Hind Hamzeh-Cognasse, Santiago Perez-Patrigeon, Jean-Daniel Lelievre, France Pirenne, Sébastien Gallien, and Benoît Vingert

---

Supplemental data

- Supplemental Table 1. Individual characteristics of PLWH.
- Supplemental Table 2. Characteristics of the PLWH population
- Supplemental Table 3. Antibody panel for flow cytometry.
- Supplemental Table 4. Real mean culture ratio by type of MPs studied.
- Supplemental Figure 1. Gating strategy and proportions of the different MPs
- Supplemental Figure 2. Gating strategy for cell and cytokine secretion analysis.
- Supplemental Figure 3. Interaction of PMPs, MMPs, LMPs with immune cells.
- Supplemental Figure 4. Cytokine secretion by different cell populations according to the co-expression of MP subpopulations between PLWH MPs and HD MPs.

<u>Supplemental Table 1</u>. Individual characteristics of PLWH

Patient ID	Sex	Age (years)	BMI (kg/m^2)	Co-infections Ab-HBV	VL-HBV	Ab-HCV	VL-HCV	Ratio CD4/CD8	Duration of HIV infection (years)	Duration of ART (years)	Type of ART
#1	M	48	26.3	pos	undetec	neg	-	1.1	22	20	3TC/ABC/DTG
#2	F	63	23.8	neg	-	pos	undetec	1.4	26	26	FTC/TAF/RPV
#3	M	55	17.7	pos	undetec	pos	undetec	2.7	36	34	RPV/DTG
#4	F	51	28.3	pos	undetec	neg	-	1.5	17	17	3TC/TDF/DOR
#5	M	70	24.8	neg	-	neg	-	0.7	35	27	DTG/ABC/3TC
#6	F	57	19.0	neg	-	neg	-	0.8	36	24	DTG/DRV/RTV
#7	M	60	19.6	pos	undetec	pos	undetec	1.4	34	25	FTC/TAF/EVG/COBI
#8	M	61	27.7	neg	-	neg	-	0.8	17	17	3TC/ABC/ATV/DTG
#9	M	66	24.3	pos	undetec	neg	-	1.7	27	26	FTC/TAF/RPV
#10	F	48	27.5	neg	-	pos	undetec	0.8	29	28	3TC/ABC/RAL
#11	F	49	30.1	neg	-	neg	-	0.5	9	9	FTC/TAF/BIC
#12	M	46	24.0	neg	-	neg	-	1.6	18	18	FTC/TAF/RPV
#13	M	63	32.5	pos	undetec	neg	-	1.4	16	15	FTC/TDF/RAL
#14	F	43	22.9	pos	undetec	neg	-	1.7	15	15	FTC/TAF/RPV
#15	F	64	33.2	neg	-	neg	-	0.7	9	9	FTC/TAF/RPV
#16	M	55	25.8	-	-	neg	-	0.7	17	14	3TC/DTG
#17	M	38	22.8	neg	-	neg	-	1.1	10	10	FTC/TAF/RPV
#18	M	57	37.0	neg	-	neg	-	1.8	8	8	3TC/DTG
#19	F	60	22.6	neg	-	neg	-	0.9	6	6	RPV/DTG
#20	M	50	31.2	pos	undetec	neg	-	1.3	14	14	RPV/CBG
#21	M	50	25.1	pos	undetec	neg	-	0.8	11	11	3TC/ABC/DTG
#22	F	64	33.3	pos	undetec	neg	-	0.8	18	17	FTC/TAF/RPV

Abbreviations: ABC abacavir, ATV atazanavir, BIC bictegravir, CBG cabotegravir, COBI cobicistat, DRV darunavir, DOR doravirine, DTG dolutegravir, EVG elvitegravir, FTC emtricitabine, RAL raltegravir, RPV rilpivirine, RTV ritonavir, TAF tenofovir alafenamide, TDF tenofovir disoproxil fumarate, 3TC lamivudine, BMI, body mass index

<u>Supplemental Table 2</u>. Characteristics of the study population

**Participant characteristics**	**PLWH (*n*=22)**
Demographics	
Male [*n* (%)]	13 (59)
Age (y) (median [min; max])	56 [38 ; 70]
BMI (kg/m^2) (median [min; max])	25 [18 ; 37]
Viral-immunological	
HIV viral load undetectable [*n* (%)]	21 (95)
$CD4^+$ T-cell count (cells/µL) (median [min; max])	707 [440 ; 1566]
Nadir $CD4^+$ T-cell count (cells/µL) (median [min;max])	180 [4 ; 727]
$CD8^+$ T-cell count (cells/µL) (median [min; max])	686 [176 ; 1386]
Duration of HIV infection (y) (median [min; max])	17 [6 ; 36]
Duration of ART (y) (median [min; max])	17 [6 ; 34]

Abbreviations: ART, antiretroviral therapy; PLWH, people living with HIV; BMI, body mass index

<u>Supplemental Table 3</u>. Antibody panel for flow cytometry

Antibody	Clone	Fluorochrome	Manufacturer
MP membrane labeling			
CD14	MφP9	BUV395	BD Biosciences $
CD3	UCHT1	BV711	BD Biosciences
CD41a	HIP8	APC-H7	BD Biosciences
Cell membrane labeling			
CD11c	BU15	BV421	BioLegend §
CD123	6H6	BV421	BioLegend
CD14	M5E2	BV650	BD Biosciences
CD3	UCHT1	BUV737	BD Biosciences
CD4	RPA-T4	PE	BioLegend
CD8	RPA-T8	BV510	BD Biosciences
Cell intracellular labeling			
IFNγ	B27	BV605	BioLegend
IL12	SNKY35	eF660	Thermo Fisher Scientific £
IL17A	N49-653	BV786	BD Biosciences
IL17F	O33-782	BV786	BD Biosciences
IL2	MQ1-17H12	PE-Dazzle594	BioLegend
IL6	MQ2-6A3	FITC	BD Biosciences
TNFα	MP6-XT22	PE-Cy7	BioLegend

$ BD Biosciences, San Jose, CA

§ BioLegend, San Diego, CA

£ Thermo Fisher Scientific, Waltham, MA

<u>Supplemental Table 4</u>. Real mean culture ratio by type of MPs studied

	Ratio PBMCs:MPs	
Sum of labeled MPs	1:1	1:20
$CD41a^+$ PMP	1:0.6	1:13
$CD14^+$ MMP	1:0.2	1:3
$CD3^+$ LMP	1:0.2	1:4

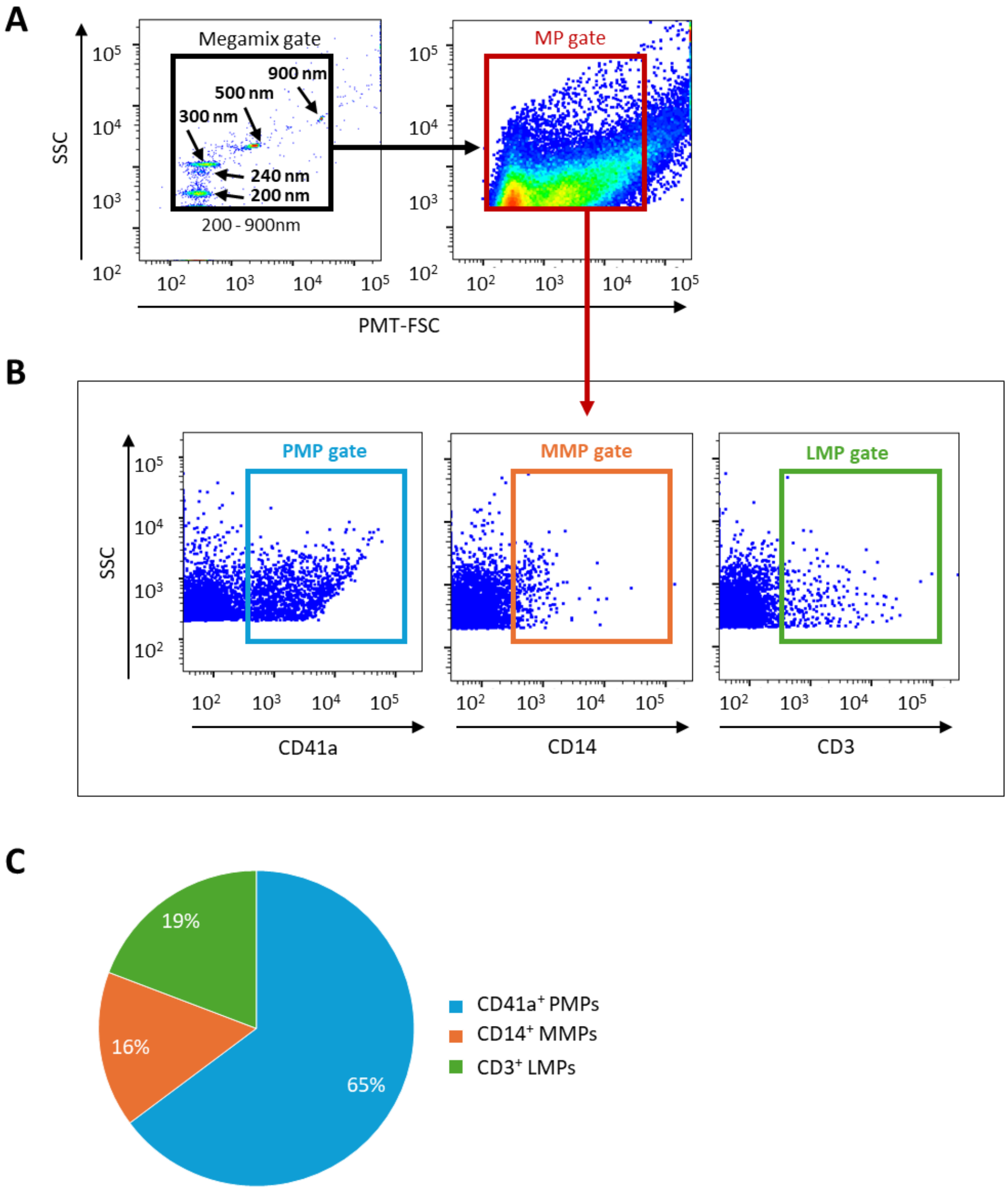

Supplemental Figure 1. **Gating strategy and proportions of the different MPs.** (**A**) Example of the gating strategy used to define MPs from PLWH by flow cytometry (*n*=22, 10 independent experiments). The dot plot on the left shows the settings based on Megamix fluorescent beads for the differentiation of particles with diameters of 200 nm, 240 nm, 300 nm, 500 nm and 900 nm. The dot plot on the right shows the MP gate used for acquisition on a Fortessa flow cytometer for one representative PLWH. (**B**) Dot plots for CD41a^{+} PMPs, CD14^{+} MMPs and CD3^{+} LMP are shown for acquisition on a Fortessa flow cytometer for one representative PLWH. Flow cytometry data were analyzed with FlowJo software. (**C**) Representation of the proportion of the three different types of MPs studied.

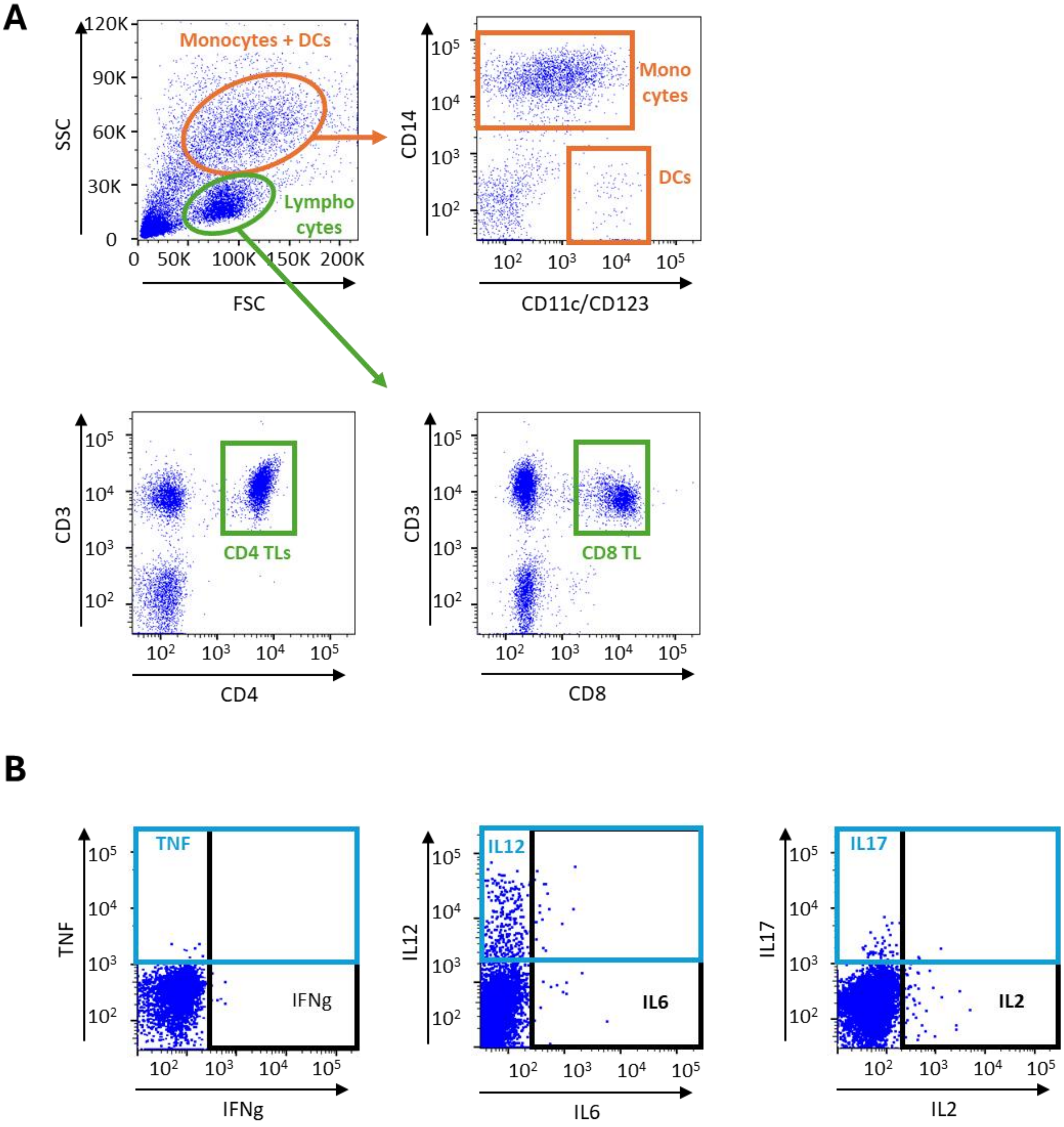

<u>Supplemental Figure 2</u>. Gating strategy for cell and cytokine secretion analysis. (**A**) Example of the gating strategy used for cell analysis by flow cytometry for one representative HD (*n*=22, 10 independent experiments). Lymphocytes, monocytes and DCs were gated on size (forward scatter, FSC) and granularity (side scatter, SSC). Monocytes and DCs were identified with the $CD14^{+}$ and $CD11c/CD123^{+}$ gates, respectively. $CD4^{+}$ and $CD8^{+}$ T lymphocytes (TL4 and TL8) were identified with the $CD3^{+}CD4^{+}$ and $CD3^{+}CD8^{+}$ gates, respectively. (**B**) Example of the gating strategy used to analyze cytokine secretion by flow cytometry for one representative HD (*n*=22, 10 independent experiments). Fluorescence was assessed on an LSR Fortessa flow cytometer, and the flow cytometry data were analyzed with FlowJo software.

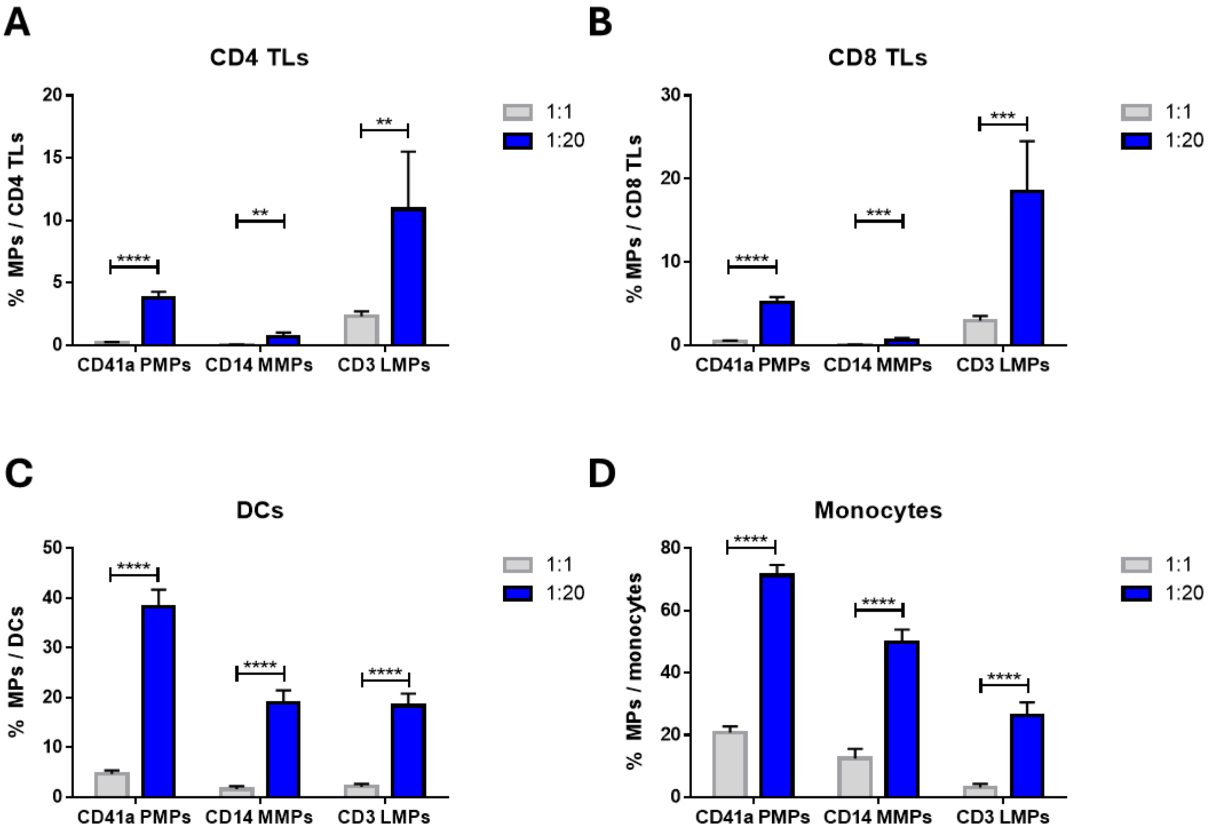

<u>**Supplemental Figure 3**</u>. **Interaction of PMPs, MMPs, LMPs with immune cells.** (**A-D**) Percentage of PLWH MPs bound to TLs ($CD4^+$ and $CD8^+$, **A-B**, respectively), DCs ($CD11c^+/CD123^+$, **C**) and monocytes ($CD14^+$, **D**) after 18 h of culture at a ratio of 1:1 or 1:20 (PBMCs: MPs), *n*=22, 10 independent experiments. *P* values were obtained in a Wilcoxon test: **** *P*<0.0001, *** *P*<0.001, ** *P*<0.01.

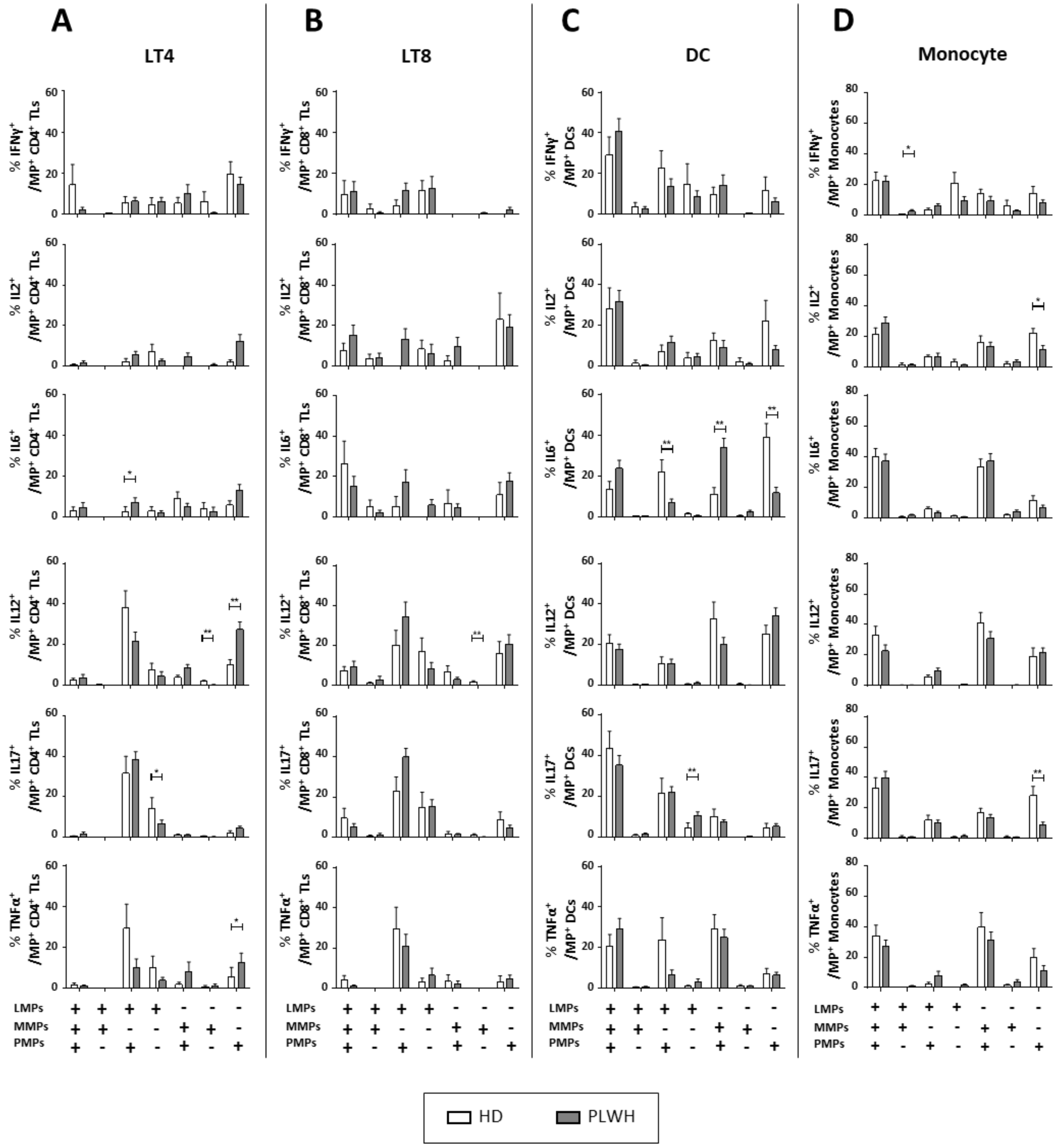

<u>Supplemental Figure 4</u>. Cytokine secretion by different cell populations according to the co-expression of MP subpopulations between PLWH MPs and HD MPs. (**A-D**) Percentage of cytokines secreted by CD4$^+$ T lymphocytes (**A**, LT4), CD8$^+$ T lymphocytes (**B**, LT8), dendritic cells (**C**, DC) and monocytes (**D**, monocyte) according to the co-expression of CD3$^+$ LMPs, CD14$^+$ MMPs and/or CD41a$^+$ PMPs from HD (white bars, *n*=10, except for TNF *n*=6, three independent coculture experiments) or PLWH (grey bars, *n*=22, except for TNF, *n*=16, 10 independent coculture experiments) at ratio 1:20 (PBMC: MPs) are expressed. *P* values (*P*<0.05 considered significant) were obtained in Mann-Whiney and post-hoc tests. ** *P*<0.01, * *P*<0.05.
